# Proteomic analysis of metronidazole resistance in the human facultative pathogen *Bacteroides fragilis*

**DOI:** 10.3389/fmicb.2023.1158086

**Published:** 2023-03-31

**Authors:** Ana Paunkov, Karin Hummel, Doris Strasser, József Sóki, David Leitsch

**Affiliations:** ^1^Institute for Specific Prophylaxis and Tropical Medicine, Center for Pathophysiology, Infectiology, and Immunology, Medical University of Vienna, Vienna, Austria; ^2^VetCore Facility for Research, University of Veterinary Medicine, Vienna, Austria; ^3^Faculty of Medicine, Institute of Medical Microbiology, University of Szeged, Szeged, Hungary

**Keywords:** *Bacteroides fragilis*, metronidazole, antimicrobial resistance, proteomics, *nimA* gene

## Abstract

The anaerobic gut bacteria and opportunistic pathogen *Bacteroides fragilis* can cause life-threatening infections when leaving its niche and reaching body sites outside of the gut. The antimicrobial metronidazole is a mainstay in the treatment of anaerobic infections and also highly effective against *Bacteroides* spp. Although resistance rates have remained low in general, metronidazole resistance does occur in *B*. *fragilis* and can favor fatal disease outcomes. Most metronidazole-resistant *Bacteroides* isolates harbor *nim* genes, commonly believed to encode for nitroreductases which deactivate metronidazole. Recent research, however, suggests that the mode of resistance mediated by Nim proteins might be more complex than anticipated because they affect the cellular metabolism, e.g., by increasing the activity of pyruvate:ferredoxin oxidoreductase (PFOR). Moreover, although *nim* genes confer only low-level metronidazole resistance to *Bacteroides*, high-level resistance can be much easier induced in the laboratory in the presence of a *nim* gene than without. Due to these observations, we hypothesized that *nim* genes might induce changes in the *B*. *fragilis* proteome and performed comparative mass-spectrometric analyses with *B*. *fragilis* 638R, either with or without the *nimA* gene. Further, we compared protein expression profiles in both strains after induction of high-level metronidazole resistance. Interestingly, only few proteins were repeatedly found to be differentially expressed in strain 638R with the *nimA* gene, one of them being the flavodiiron protein FprA, an enzyme involved in oxygen scavenging. After induction of metronidazole resistance, a far higher number of proteins were found to be differentially expressed in 638R without *nimA* than in 638R with *nimA*. In the former, factors for the import of hemin were strongly downregulated, indicating impaired iron import, whereas in the latter, the observed changes were not only less numerous but also less specific. Both resistant strains, however, displayed a reduced capability of scavenging oxygen. Susceptibility to metronidazole could be widely restored in resistant 638R without *nimA* by supplementing growth media with ferrous iron sulfate, but not so in resistant 638R with the *nimA* gene. Finally, based on the results of this study, we present a novel hypothetic model of metronidazole resistance and NimA function.

## 1. Introduction

The genus *Bacteroides* comprises several of the most common anaerobic bacteria in the human intestine and accounts for 30% of the human fecal isolates (Kuwahara et al., 2004). Their role is predominantly beneficial as they break down carbohydrates which would otherwise be inaccessible to the human host. Thereby they produce volatile short-chain fatty acids such as succinic acid which are absorbed in the gut (Wexler, 2007). *Bacteroides* spp., however can also cause severe disease when the integrity of the intestinal wall is compromised, e.g., through cancer or injuries, and they can leave their intestinal niche to cause abscesses in other body sites or bacteremia (Aldridge and Sanders, 2002; Wexler, 2007). This is especially true for *Bacteroides fragilis* which accounts only for 1% of the intestinal microbiome but for as much 30%–60% of all anaerobic clinical isolates in total (Wexler, 2007; Tan et al., 2017). Importantly, *B*. *fragilis* secretes fragilysin, a metalloprotease which digests E-cadherin and can disrupt the tight junctions of the intestinal epithelium (Remacle et al., 2014).

*Bacteroides* infections are commonly treated with carbapenems and metronidazole. Metronidazole is a 5-nitroimidazole drug specifically targeting anaerobic and microaerophilic microbes (reviewed in Leitsch, 2019). The specificity of metronidazole for anaerobes and microaerophiles is due to a requirement for the reduction of the nitro group, which only takes place quantitatively in low oxygen environments. The resulting reactive intermediates damage the cell through forming adducts with proteins, inducing DNA strand breaks and by depleting cellular redox buffers (Leitsch, 2019). Arguably, either the nitroimidazole anion, resulting from a single electron transfer to the nitro group, or the nitroso radical, resulting from a double electron transfer to the nitro group, are causing most of the damage. In contrast to many other antibiotics, metronidazole has retained usability due to overall low resistance rates, especially in true anaerobes such as *Bacteroides* spp. (Snydman et al., 2017). However, metronidazole resistance in *Bacteroides* spp. does occur and can be found in a sizeable proportion of isolates in certain areas (Vieira et al., 2006; Yehya et al., 2014; Sheikh et al., 2015).

Metronidazole resistance in the field is mainly but not exclusively observed in isolates carrying *nim* genes. To date, 12 different homologs of *nim* were detected in *Bacteroidetes* (*nimA*-*nimL*), all of which are believed to encode nitroreductases capable of transferring six electrons to the nitro group of metronidazole, resulting in a non-toxic amine (reviewed in Alauzet et al., 2019). Although there are data supporting this hypothesis (Carlier et al., 1997), a direct mode of Nim proteins as nitroreductases has been questioned by other findings (Leitsch et al., 2014; Paunkov et al., 2022a). Importantly, Nim expression levels are widely independent of the degree of metronidazole resistance (Leitsch et al., 2014; Paunkov et al., 2022a) although the proposed role of Nim proteins as nitroreductases would logically imply a positive correlation of expression levels and the degree of metronidazole resistance. Such positive correlation, however, could be only observed in the initial stage of resistance as conferred by *nim* genes (Kupc et al., 2022), but not after adaptation to higher, medically relevant, doses of the drug (4 μg mL^−1^ according to EUCAST, see https://www.eucast.org/clinical_breakpoints). Furthermore, *nim* genes are positively correlated to a greatly increased activity of pyruvate:ferredoxin oxidoreductase (PFOR; Paunkov et al., 2022a), a central metabolic enzyme in most anaerobes, including *B*. *fragilis* (Narikawa, 1986). This is surprising given that PFOR is commonly believed to be a major metronidazole-reducing enzyme (Narikawa, 1986) and therefore a source of reactive metronidazole intermediates. After induction of high-level metronidazole resistance in the laboratory, i.e., adaptation to high concentrations of metronidazole, PFOR activity is retained in *B*. *fragilis* 638R with the *nimA* gene, but lost without it (Paunkov et al., 2022a). Moreover, *B*. *fragilis* 638R carrying the *nimA* gene also retains good tolerance to oxygen after induction of high-level metronidazole resistance, whereas as metronidazole-resistant 638R without the *nimA* gene becomes highly sensitive to oxygen (Paunkov et al., 2022a).

These observations suggest that the development of high-level metronidazole resistance is strongly facilitated in the presence of the *nimA* gene and might be caused by a different mechanism than observed in strains without the *nimA* gene. In order to address this question and to identify differentially expressed proteins we analyzed and compared the proteomes of *B*. *fragilis* 638R without a *nim* gene and of *B*. *fragilis* 638R harboring *nimA* by quantitative mass spectrometry before and after induction of high-level metronidazole resistance.

## 2. Materials and methods

### 2.1. Chemicals and growth media components

Vitamin K1, brain-heart-infusion broth (BHI), and agar were purchased from Carl Roth (Karlsruhe, Germany). Wilkins-Chalgren anaerobe agar (WC) was purchased from Oxoid (Basingstoke, England). Hemin was purchased from Sigma-Aldrich (St. Luis, United States) and Anaerocult A was purchased from Merck (Darmstadt, Germany). Metronidazole was purchased from Sigma-Aldrich (St. Luis, United States), and Etests were purchased from bioMérieux (Marcy-l’Étoile, France).

### 2.2. Bacterial strains and culture

The experiments were performed with *B*. *fragilis* 638R (division I, *cfiA*-) and a transconjugant daughter strain with plasmid pI417 harboring the *nimA* gene (Breuil et al., 1989). The MICs for metronidazole according to Etests were: 638R, 0.25 μg mL^−1^ (Paunkov et al., 2022b); and 638R *nimA*, 1.5 μg μg mL^−1^ (Paunkov et al., 2022b). Importantly, the breakpoint concentration for metronidazole resistance amounts to 4 μg mL^−1^ according to EUCAST, 2023. The *nimA* gene is positioned behind insertion element IS1168 (Haggoud et al., 1994) whose sequence can be accessed *via* GenBank number X71444. Both strains were obtained from the strain repository of the Institute of Medical Microbiology in Szeged, Hungary. The genome sequence of 638R can be accessed in the NCBI database *via* accession number FQ312004. Induction of high-level resistance was performed as described previously (Paunkov et al., 2022a) by passaging 638R and 638R *nimA* with increasing metronidazole concentrations in the agar (factor 2 with every passage). The induction process was terminated after having achieved growth at a metronidazole concentration of 64 μg mL^−1^. The four strains, i.e., 638R and its three derivatives, are designated as 638R, 638R^R^, 638R *nimA*, and 638R *nimA*^R^, respectively, throughout the manuscript, “R” indicating “resistant.”

Cells were grown on either WC agar plates or BHI agar plates with hemin and vitamin K1 supplementation. When cultures were prepared for mass spectrometry, cells were grown anaerobically at 37°C in BHI broth (Carl Roth, Germany) supplemented with 1 μg mL^−1^ vitamin K1 and 5 μg mL^−1^ hemin (BHI-S) inside 2.5 L anaerobic jars (Merck, Darmstadt, Germany) conditioned with the Anaerocult A system (0% O_2_ and 18% CO_2_).

### 2.3. RT-qPCR

RT-qPCR was performed exactly as described recently (Paunkov et al., 2022a) with *rpoD* and *gapdh* mRNA as internal standards for normalization. The primers for the amplification of a 176 nt fragment within the flavodiiron protein A (*fprA*) cDNA were 5′-CAATGTCAGCAAAAGTAATCCC-3′ (forward) and 5′-GTGAACGAACCGAAATACCC-3′ (reverse).

### 2.4. Episomal expression of *fprA* in *Bacteroides fragilis* 638R

PCR amplification of *fprA* gene was performed using forward 5′-GAG GAT CCA TGG AAC AGA AAA CAA GAA TTA AAG G-3′ and reverse 5′-GAG GTA CCT TAT ACT CTG TCT TTT TTT AAA CGG C-3′ primers containing *Bam*HI and *Kpn*I restriction sites, respectively. The gene was inserted into the pFD340 vector with the same restriction sites. The resulting pFD*fprA* plasmid was introduced into *E. coli* 10-beta cells using the manufacturer’s instructions. Transformed cells were selected on LB plates with 100 μg ml^−1^ ampicillin. An established tri-parental filter mating protocol (Mays et al., 1982), using *E*. *coli* RK231 as a helper strain, was applied to transfer plasmids from *E. coli* 10-beta to *B*. *fragilis* 638R. Briefly, *E. coli* 10-beta and *E. coli* RK231 cells were grown on LB plates with 100 μg mL^−1^ ampicillin and 150 μg mL^−1^ streptomycin and spectinomycin, respectively. *Bacteroides fragilis* 638R cells were grown on BHI-S plates with 20 μg mL^−1^ rifampicin. A single colony of each strain was grown with antibiotics overnight at 37°C then diluted 1:100 (*E. coli* strains) or 1:40 (*B*. *fragilis* 638R) and further grown in antibiotic-free medium until an OD_600_ of 0.5 was reached. 1 mL of *B*. *fragilis* 638R and 0.25 mL of each *E. coli* culture were united in sterile centrifuge tubes, and centrifuged for 30 s at maximum speed. The resulting pellet was resuspended in 100 μL of BHIS medium and transferred onto sterile filters placed on mating plates which had been previously incubated overnight inside an anaerobic chamber at 37°C. Plates with filters carrying a mixture of all three strains were then incubated overnight at 37°C aerobically. Cells were resuspended by vortexing the filters for 1 min inside 15 mL falcon tubes in 1 ml of BHIS medium. Conjugants were grown and selected on BHIS plates with 10 μg mL^−1^ erythromycin, 20 μg mL^−1^ rifampicin, and 100 μg mL^−1^ gentamycin after 48 h of anaerobic incubation at 37°C.

### 2.5. Oxygen scavenging measurements

2 × 10^9^ cells were harvested from an overnight culture (3,000 × *g* for 5 min) and suspended in 14 mL of BHI medium which had previously been saturated with oxygen through vigorous shaking. Cells were then transferred into a 15 ml glass tube and an oxygen microsensor (OX-500, Unisense, Denmark) of an oximeter (OXY-Meter, Unisense, Denmark) was inserted. In order to prevent inflow of air, the glass tube was sealed with Parafilm M. Oxygen concentrations were measured for 60 min with automatic recording of values every 30 s. Oxygen removal by the components of the medium was also measured in the same setup in the absence of cells. These background values were subtracted from the values obtained in the presence of cells.

### 2.6. Susceptibility testing

Susceptibility tests were performed by picking colonies with a sterile swab and resuspending them in 1× PBS before application on BHI agar plates (either with or without supplementation with ferrous iron sulfate). Plates were left for 15 min to dry and Etests were applied. Plates were transferred to anaerobic jars and incubated for 48 h at 37°C. The MICs were read at the intersection of the border of growth inhibition and the test strip. Etest images were obtained using BioRad GelDoc XR.

### 2.7. Sample preparation and protein analysis by nanoHPLC-MS/MS

For protein isolation, 4 × 10^9^ cells from an overnight culture grown in BHI-S broth were harvested by centrifugation at 3,000 × *g* for 10 min. After centrifugation, the medium was removed and cells were washed with 1 ml ddH_2_O and centrifuged once again at 3,000 × *g* for 10 min. The supernatant was removed and cells were resuspended in 120 μL denaturing protein sample buffer [7 M urea, 2 M thiourea, 1% DTT, 1% Biolyte^®^ ampholytes (BioRad), 4% CHAPS] and 1 μL of Turbonuclease^®^ from *Serratia marcescens* (Sigma-Aldrich) was added for the removal of genomic DNA in the samples. Cells were then sonicated at 4°C (10 cycles 30 s pulse on, and 30 s pulse off) and centrifuged for 20 min at 4°C and 20,000 × *g*. The resulting supernatant was transferred into fresh tubes, and protein concentrations were determined using Bradford assay. For protein digestion, sample extracts were prepared with a filter-aided sample preparation protocol based on Wisniewski et al. (2009) and Wisniewski (2016). In brief, 30 μg protein solved in lysis buffer was filled up to 500 μL with 8 M Urea in 50 mM Tris buffer (both Roth, Karlsruhe, Germany) and loaded onto an Pall 10 kDa ultrafiltration device (Pall Corporation, Port Washington, NY, United States). After centrifugation (20 min at 10,000 × *g*), cysteine residues were reduced with 200 mM DTT (37°C, 30 min) and alkylated with 500 mM iodoacetamide (37°C, 30 min) on the filter (both Sigma-Aldrich, St. Louis, MO, United States). This was followed by two washes with 100 μL 50 mM Tris before digestion with trypsin/LysC Mix for 14 h (Promega Technical Manual, Madison, WI, United States). Digested peptides were recovered with 3 times 50 μL of 50 mM Tris buffer. Before LC-MS analysis, acidified peptide extracts were purified using C18 spin columns (Pierce, Thermo Fisher Scientific) according to the manufacturer’s protocol. Dried peptides were redissolved in 300 μL 0.1% trifluoroacetic acid (Thermo Fisher Scientific, Waltham, MA, United States) and 300 ng digested protein was injected into the nanoRSLC-nESI-QExactive-Orbitrap HF MS/MS system (Thermo Fisher Scientific). Mass spectrometry analysis was performed as described in Gutiérrez et al. (2019).

### 2.8. Statistical evaluation of nanoHPLC-MS/MS data

For the label-free quantitative shotgun proteomic approach, mass spectrometry analysis was done in two technical replicates per biological replicate, i.e., six replicates in total per isolate. Evaluation of raw data was accomplished with the software Proteome Discoverer version 2.4.1.15. (Thermo Fisher Scientific). A combination of the *Bacteroides fragilis* database downloaded from RefSeq (taxonomy 817, https://www.ncbi.nlm.nih.gov/refseq/, accessed on 24 October 2022) and a common contaminant database (https://www.thegpm.org/crap/, accessed on 25 June 2019) was used. Furthermore, the sequence of the nitroimidazole resistance protein NimA (WP_032488596.1 from *Phocaeicola vulgatus*) was added. Search parameters were applied as follows: trypsin as an enzyme; maximally 2 missed cleavages; 10 ppm precursor mass tolerance and 0.02 Da fragment mass tolerance; dynamic modifications allowed were oxidation/+15.995 Da (M)/deamidation/+0.984 Da (N, Q)/Gln->pyro-Glu/−17.027 Da (Q)/acetylation/+42.011 Da (N-Terminus)/methionine loss/−131.040 Da (M) and static modification carbamidomethylation/+57.021 Da (C).

For intensity-based label-free quantification (LFQ), protein abundance raw values were generated in Proteome Discoverer software including normalization to total area sums. Further ANOVA analysis was performed in R version 4.0.5 (R Core Team, 2021). Prior to data import into R, protein abundances of technical replicates were aggregated by the mean. Furthermore, proteins with at least one missing value per group were excluded from the quantification analysis, in order to keep so-called “ON/OFF” proteins, but maintain high data quality. Statistical analysis by ANOVA depending on the number of groups was performed in R Studio. Proteins recognized with more than two tryptic peptides and displaying a fold change higher/lower than +/−2-fold with an adjusted *value of p* for controlling the false discovery rate according to Benjamini-Hochberg lower than 0.05, were considered to be upregulated/downregulated.

### 2.9. Statistical analysis

Statistical tests were performed with GraphPad Prism 9 software. More details are given in the respective table and figure legends.

## 3. Results

### 3.1. Quantitative mass-spectrometric analysis of strain 638R without a *nim* gene and of 638R with the *nim*A gene, before and after induction of high-level metronidazole resistance

In a previous study, we had shown that strain *B*. *fragilis* 638R displayed elevated PFOR activity (Paunkov et al., 2022a) when the *nimA* gene was present. The *nimA* gene confers reduced susceptibility to metronidazole, with an MIC of approximately 1.5 μg mL^−1^ as compared to an MIC of 0.25 μg mL^−1^ in 638R wildtype (Paunkov et al., 2022b). We had also observed that the development of high-level metronidazole resistance (≥ 64 μg μg mL^−1^) proceeds much faster and does not entail grave physiological impairments such as the loss of PFOR activity or high sensitivity to oxygen when *nimA* was present (Paunkov et al., 2022a). Hence, we speculated that high-level metronidazole resistance was caused by different mechanisms in 638R wildtype (henceforth designated as 638R) and in 638R with a *nimA* gene (henceforth designated as 638R *nimA*), and that this would be mirrored by differential expression of relevant proteins. In order to address this hypothesis, we subjected cell extracts of strain 638R and of 638R *nimA* to proteomic analyses using mass spectrometry. The analysis was performed twice at two different time points in order to ensure reproducibility of the results. Further, also highly metronidazole-resistant derivatives of 638R (henceforth designated as 638R^R^) and of 638R *nimA* (henceforth designated as 638R *nimA*^R^), both resistant to at least 64 μg mL^−1^ of metronidazole, were analyzed by mass spectrometry and compared to the original strains. The highly metronidazole-resistant cell lines had been generated in a recent study (Paunkov et al., 2022a) by growing cells on increasing concentrations of metronidazole with each subculture. The mass spectrometric data obtained were deposited at the PRIDE database (Perez-Riverol et al., 2022).

#### 3.1.1. *Comparison of 638R* vs. *638R* nimA

First, it was assessed if the presence of *nimA* induces changes in the protein expression profile as compared to the 638R wildtype. Protein numbers in all samples (biological triplicates in technical duplicates) ranged from about 1,000 to 1,500. Only changes in expression of at least twofold and at a significance level of *p* < 0.01 were considered. Further, only proteins of which peptides were found in all samples were considered. The first analysis with 638R *nimA* gave 24 differentially expressed proteins as compared to 638R (Table 1; Supplementary Table 1), whereas the second analysis gave 46 differentially expressed proteins (Table 1; Supplementary Table 2). However, although the number of differentially expressed proteins was similar, only five proteins were shared in both data sets (Table 2). Of these, only one protein was downregulated, i.e., exo-alpha-sialidase (WP_005794995), the other four were upregulated. One of the upregulated proteins was galactokinase (8.6-fold upregulated in the first run, and 12.5-fold in the second) and another was type A flavoprotein (FprA; WP_005785280), a flavodiiron protein commonly found in bacteria (Wasserfallen et al., 1998) which reduces either molecular oxygen to water or nitric oxide (NO) to nitrous oxide (N_2_O; Martins et al., 2019). FprA was upregulated 3.7-fold in the first run, and 12.9-fold in the second.

**Table 1:** The mass-spectrometric analyses performed.

Strain	Differentially expressed proteins as compared to 638R
638R *nimA* (first run)	24
638R *nimA* (second run)	46
638R^R^	237
	Differentially expressed proteins as compared to 638R *nimA*
638R *nimA*^R^	41

**Table 2:** Proteins consistently upregulated or downregulated in 638R *nimA* vs. 638R in two independent mass-spectrometric analyses.

Name	Gene ID	Prot. ID analysis	-fold up (+) or down (−)	-fold up (+) or down (−)
			First run	Second run
FprA family A-type Flavoprotein	BF638R_RS04550	WP_005785280	+3.7	+12.9
	BF638R_0963			
Galactokinase	BF638R_RS07740	WP_005786505	+8.6	+12.5
	BF638R_1663			
Exo-alpha-sialidase	BF638R_RS08060	WP_005794995	−3.1	−2.8
	BF638R_1728			
Dihydrofolate reductase Family protein	BF638R_RS10400	WP_014298858	+2	+2
	BF638R_2202			
1-deoxy-D-xylulose-5-phosphate synthase	BF638R_RS20210	WP_042987589	+2.2	+2.4
	BF638R_4171			

#### 3.1.2. *Comparison of 638R* vs. *638R^R^ and of 638R* nimA vs. 638R nimA^R^

Next, we analyzed the proteomes of 638R^R^ and 638R *nimA*^R^ and compared them with their respective non-resistant parent strains. Applying the same selection criteria as stated above, 41 proteins were found to be differentially expressed in 638R *nimA^R^* as compared to the non-resistant parent 638R *nimA* (Table 1; Supplementary Table 3). Only eight of the 41 proteins were upregulated in expression, seven of which are obviously organized in an operon for nucleotide sugar synthesis (WP_005801381, WP_032600526, WP_100766060, WP_005784923, WP_005784927, WP_005784941, and WP_014298264). Downregulated enzymes included aminotransferases, N-acetyl transferases, enzymes involved in capsule synthesis (Zhang et al., 2003; WP_005817136, WP_014298686), and numerous other factors whose correlation with metronidazole resistance remained unclear. None of the five proteins found to be differentially upregulated in 638R *nimA* as compared to 638R were further upregulated or downregulated, respectively, in 638R *nimA*^R^.

**Figure 1:**
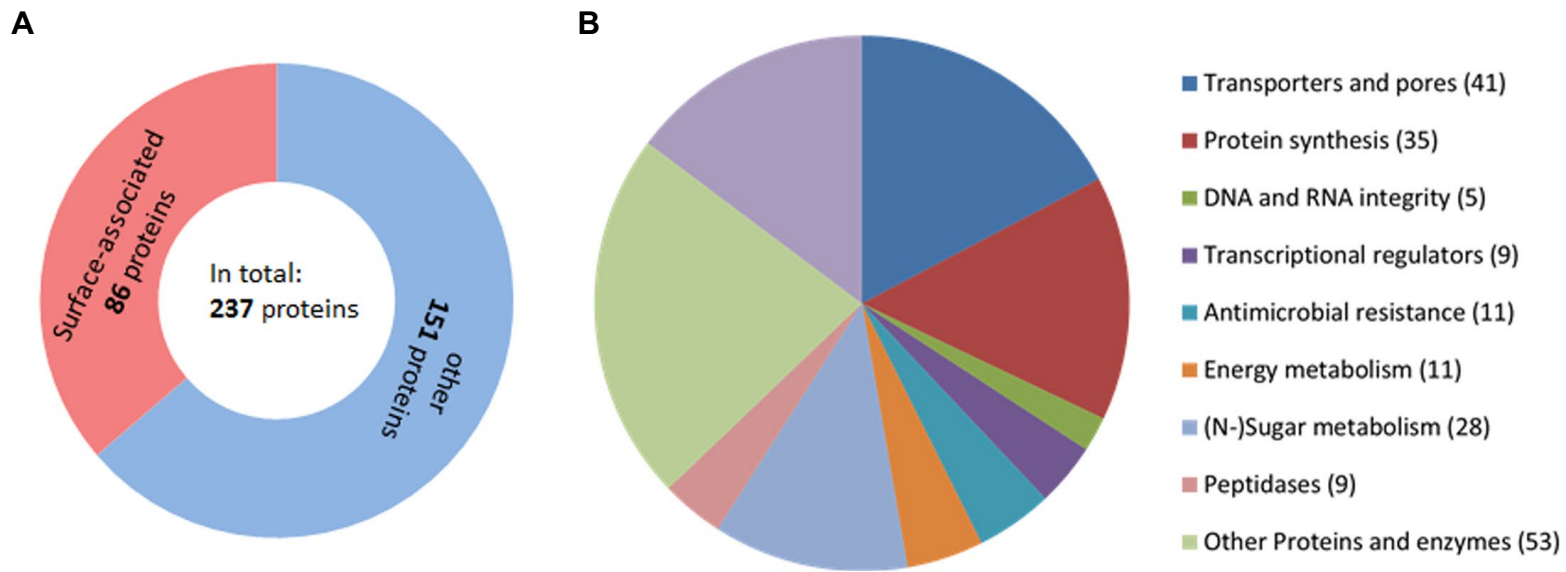
Proteins identified to be differentially expressed in *Bacteroides fragilis* 638R^R^ as compared to 638R. **(A)** Total number of proteins differentially expressed as compared to wildtype 638R and their cellular localization. **(B)** Pie chart indicating the functions of the proteins differentially expressed in 638R^R^.

In 638R^R^, a comparably large number of proteins, i.e., 237, were found to be differentially expressed (Table 1; Supplementary Table 4) as compared to wildtype 638R. The levels of most differentially expressed proteins were increased (189) rather than decreased (48), and a very large proportion of the proteins, i.e., at least 84 of 237, are likely or known to be surface-associated, 41 being transporters or pores (Figure 1; Supplementary Table 5). When comparing 638R^R^ to 638R *nimA*, three of the five proteins previously identified to be consistently differentially expressed in 638R *nimA* as compared to 638R were also found to be differentially expressed in 638R^R^ (Supplementary Table 4). Two of these, however, exo-alpha-sialidase and 1-deoxy-D-xylulose-5-phosphate, were inversely expressed in the two strains, i.e., upregulated in one and downregulated in the other. Only FprA was upregulated in 638R *nimA* and 638R^R^ alike. When comparing 638R^R^ to 638R *nimA*^R^, 19 proteins were found differentially expressed in both strains (Supplementary Table 4). Eleven of these proteins, however, were upregulated in one strain but downregulated in the other, suggesting that they are not inherently related to metronidazole resistance. Of the other eight proteins, all were consistently downregulated in both strains. Five of these are putatively involved in carbohydrate metabolism (WP_005801178, WP_005795861, WP_008768174, WP_005794845, and WP_005817136), one is a hypothetical protein (WP_032542280), another an acetyl transferase (WP_032531450), and finally, HemY (WP_005791059) is a protoporphyrinogen oxidase which is tentatively involved in heme synthesis.

Importantly, several proteins involved in the import or synthesis of heme were found differentially expressed in 638R^R^ as compared to 638R (Table 3; Supplementary Tables 4, 5). Apart from HemY, two homologs of heme-binding protein HmuY (WP_032556399 and WP_010993105), their cognate TonB-dependent receptors (WP_032564333 and WP_005788361), and a ChaN-like lipoprotein, presumably involved in heme uptake (Chan et al., 2006), were all strongly downregulated. One of the HmuY homologs, WP_032556399, had also recently been found downregulated at the mRNA level in 638R^R^ (Paunkov et al., 2022a). In contrast, several enzymes dependent on heme or iron were upregulated in 638R^R^ (Table 3; Supplementary Tables 4, 5): catalase, cytochrome ubiquinol oxidase subunit I, and fumarate reductase cytochrome b subunit. Further, also NADH:ubiquinone reductase subunits B, C, and F were upregulated. NADH:ubiquinone reductase feeds electrons into the electron transport chain which are used downstream by fumarate reductase and cytochrome ubiquinol oxidase in the reduction of fumarate or oxygen, respectively (Baughn and Malamy, 2004; Franke and Deppenmeier, 2018; Butler et al., 2022). In addition to fumarate reductase, also three other metabolic enzymes, i.e., pyruvate phosphate dikinase (PPDK), acetyl-CoA hydrolase, and butyrate kinase, were upregulated (Table 3). Pyruvate formate-lyase (PFL), an important metabolic enzyme which breaks down pyruvate to acetyl-CoA and formate, however, was strongly downregulated (Table 3).

**Table 3:** Proteins involved in heme import, antioxidant defense, and energy metabolism differentially expressed in 638R^R^ vs. 638R.

Name	Gene ID	Protein ID	-fold up (+) or downregulation (−)
**Heme import and synthesis**			
HmuY family protein	BF638R_RS05165	WP_032556399	−509.4
	BF638R_1099		
TonB-dependent receptor	BF638R_RS05170	WP_032564333	−77.2
	BF638R_1100		
HmuY family protein	BF638R_RS12955	WP_010993105	−19.9
	BF638R_2716		
TonB-dependent receptor	BF638R_RS12960	WP_005788361	−10.8
	BF638R_2717		
Protoporphyrinogen oxidase	BF638R_RS18075	WP_005791059	−5.3
	BF638R_3760		
ChaN family lipoprotein	BF638R_RS16635	WP_005790492	−7.2
	BF638R_3469		
**Antioxidant defense**			
Cytochrome ubiquinol oxidase subunit I	BF638R_RS08960	WP_032556626	+8.3
	BF638R_1909		
Catalase	BF638R_RS05930	WP_234077047	+2.1
	BF638R_1262		
**Energy metabolism**			
Acetyl-CoA hydrolase/transferase family protein	BF638R_RS00125	WP_005783582	+2
	BF638R_0025		
Pyruvate formate-lyase-activating protein	BF638R_RS06275	WP_032593684	−10.8
	BF638R_1338		
Formate C-acetyltransferase	BF638R_RS06280	WP_005786073	−5.1
	BF638R_1339		
NADH:ubiquinone reductase (Na(+)-transporting) subunit B	BF638R_RS10070	WP_025814654	+8.5
	BF638R_2137		
Na(+)-translocating NADH-quinone reductase subunit C	BF638R_RS10075	WP_005787203	+5.5
	BF638R_2138		
NADH:ubiquinone reductase (Na(+)-transporting) subunit F	BF638R_RS10090	WP_005800144	+3.6
	BF638R_2141		
Pyruvate, phosphate dikinase	BF638R_RS12250	WP_032595946	+2.1
	BF638R_2565		

Thirty-five proteins involved in protein synthesis, i.e., 31 ribosomal proteins, translation initiation factor IF-3, RluA family pseudouridine synthase, elongation factor G, and pseudouridine synthase were upregulated (Supplementary Tables 4, 5), while none were downregulated in 638R^R^. Further, a subset of 11 surface-associated proteins which are assumed or known to be involved in antimicrobial resistance were upregulated (Table 4; Supplementary Tables 4, 5): an ABC transporter ATP-binding protein, an aminoglycoside phosphotransferase, an MBL fold metallo-hydrolase, a TolC protein, and nine components of RND (Resistance-Nodulation-Division) family transporters. The latter are of special significance as they had been reported before to be associated with metronidazole resistance (Pumbwe et al., 2006, 2007). Indeed, eight of the nine could be matched in the NCBI database to BmeA2 and BmeB2, BmeA5 and BmeB5, BmeA13 and BmeB13, and BmeA15 and BmeB15. Of these, especially BmeA5 and BmeB5 have been implied to be involved in metronidazole resistance as part of the BmeRABC5 multidrug efflux system (Pumbwe et al., 2007), but also BmeB2 had been found upregulated in *B*. *fragilis* strains after selection for metronidazole resistance (Pumbwe et al., 2006).

**Table 4:** Putative antibiotic resistance factors differentially expressed in 638R^R^ vs. 638R.

Name	Gene ID	Protein ID	-fold up (+) or downregulation (−)
ABC transporter ATP-binding protein	BF638R_RS00810	WP_005813825	+11.5
	BF638R_0178		
Efflux RND transporter periplasmic adaptor subunit	BF638R_RS02635	WP_005784439	+8.7
	BF638R_0554		
Aminoglycoside phosphotransferase family protein	BF638R_RS04280	WP_032532743	+2.1
	BF638R_0908		
Efflux RND transporter periplasmic adaptor subunit	BF638R_RS10850	WP_032529434	+3.8
*Homolog of BmeB13 (GenBank: AUI46486)*	BF638R_2297		
Efflux RND transporter permease subunit	BF638R_RS10855	WP_005787504	+4.8
*Homolog of BmeA13 (GenBank: AUI46487)*	BF638R_2298		
efflux RND transporter periplasmic adaptor subunit	BF638R_RS11550	WP_005803571	+9.7
*Homolog of BmeB2 (GenBank: AUI46629)*	BF638R_2435		
efflux RND transporter permease subunit	BF638R_RS11555	WP_032544118	+8.6
*Homolog of BmeA2 (GenBank: AUI46630)*	BF638R_2436		
MBL fold metallo-hydrolase	BF638R_RS12530	WP_005788177	+15
	BF638R_2622		
TolC family protein	BF638R_RS14315	WP_005802959	+4.7
	BF638R_2994		
Efflux RND transporter periplasmic adaptor subunit	BF638R_RS15185	WP_022347707	+8.4
*Homolog of BmeA15 (GenBank: AUI49220)*	BF638R_3174		
Efflux RND transporter permease subunit	BF638R_RS15190	WP_005789587	+2.1
*Homolog of BmeB15 (GenBank: AUI47384)*	BF638R_3176		
Efflux RND transporter permease subunit	BF638R_RS19360	WP_005797651	+5
*Homolog of BmeB5 (GenBank: AUI48178)*	BF638R_3999		
Efflux RND transporter periplasmic adaptor subunit	BF638R_RS19365	WP_005791602	+9.1
*Homolog of BmeA5 (GenBank: AUI48179)*	BF638R_4000		

Finally, a large number of transporters, channels, peptidases, nucleases, transcriptional regulators, and enzymes involved in carbohydrate metabolism were differentially expressed in 638R^R^ as compared to 638R (Supplementary Tables 4, 5).

### 3.2. Overexpression of *fprA* in *Bacteroides fragilis* 638R does not confer resistance to metronidazole

**Figure 2:**
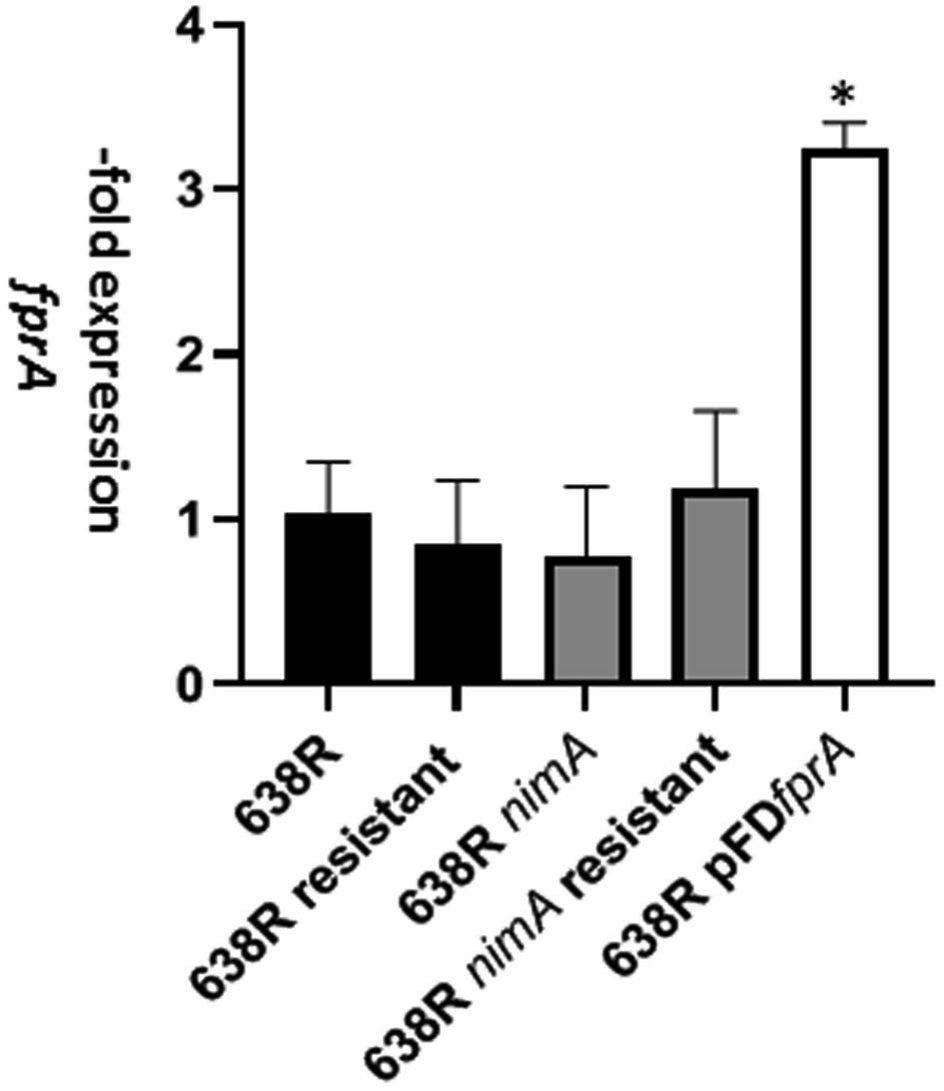
Levels of *fprA* mRNA as determined by RT-qPCR in 638R, 638R^R^, 638R *nimA*, 638R *nimA^R^*, and 638R pFD*fprA* which carries an additional copy of the *fprA* gene on shuttle plasmid pFD340 under transcriptional control of insertion element 4351 (IS*4351*). Measurements were performed in three independent experiments each. The asterisk indicates 638R pFD*fprA* > 638R with *p* < 0.001 according to Brown-Forsythe and Welch ANOVA tests with a post-hoc two stage linear step-up procedure of Benjamini, Krieger, and Yekutieli.

The flavodiiron protein FprA was found to be more strongly expressed in 638R *nimA* and in 638R^R^ as compared to 638R (Supplementary Tables 1, 2, 4). Flavodiiron proteins are a class of enzymes often found in anaerobes (Martins et al., 2019) and are important factors in the antioxidant and/or antinitrosative defense by reducing oxygen to water and/or nitric oxide (NO) to nitrous oxide (N_2_O), respectively. In order to test if overexpression of FprA conferred enhanced tolerance to metronidazole, we cloned the *fprA* gene into the pFD340 shuttle vector (Smith et al., 1992) and introduced the resulting plasmid pFD*fprA* into 638R by tri-parental mating. Subsequent analysis by RT-qPCR showed that the level of *fprA* was approximately 3-fold higher in the pFD*fprA* transconjugant than in 638R wildtype (Figure 2). Despite this elevated level of *fprA* mRNA, however, 638R pFD*fprA* displayed an even lower MIC of metronidazole as determined by Etest (Supplementary Figure 1) than the 638R parent, i.e., 0.125 μg mL^−1^ as compared to 0.25 μg mL^−1^ (Paunkov et al., 2022b). We asked if a 3-fold overexpression of *fprA* mRNA would be insufficient to obtain a measurable effect on the MLC of metronidazole and if *fprA* mRNA levels would be even higher in 638R *nimA* and 638R^R^. The levels of *fprA* mRNA in both strains, however, proved to be unchanged as compared to 638R wildtype (Figure 2), indicating that regulation of FprA expression occurs at the translational rather than the transcriptional level.

### 3.3. 638R^R^ and 638R *nimA*^R^ display impaired oxygen scavenging capacity

FprA had already been previously described in *B*. *fragilis* (Meehan et al., 2012) and in *Bacteroides thetaiotaomicron* (Mishra and Imlay, 2013), albeit under different names, i.e., Oxe and Roo, respectively. In *B*. *thetaiotaomicron,* the deletion of the *roo* gene strongly reduces oxygen scavenging capability (Lu and Imlay, 2017), suggesting that also *B*. *fragilis* FprA acts as an oxygen reducing enzyme. As such, it has the same function as cytochrome ubiquinol oxidase, also commonly termed cytochrome bd oxidase (Baughn and Malamy, 2004). The latter, however, is membrane-localized and harnesses electrons from the quinone pool, whereas flavodiiron proteins are cytoplasmic and derive electrons from reductants such as rubredoxin (Martins et al., 2019). Ultimately, both oxygen scavenging enzymes depend on NAD(P)H oxidation and have similar turnovers. In a previous study, we found that oxygen scavenging capacity in 638R *nimA* is slightly but significantly enhanced as compared to 638R (see Supplementary Figure 1 in Paunkov et al., 2022b) which is in accordance with the higher levels of FprA in the former (Table 2). We asked how oxygen scavenging rates would develop in the course of development of metronidazole resistance and measured oxygen scavenging rates in 638R^R^ and 638R *nimA*^R^. These proved to be approximately halved in both strains and were not significantly different from each other (Table 5). At least for 638R *nimA*^R^, this result suggests that either less reductant, i.e., NADH or NADPH, is available for oxygen scavenging or that cytochrome ubiquinol oxidase is less active. An impairment of FprA-mediated oxygen scavenging can be ruled out because the FprA expression level is equal to that in 638R *nimA* and good supply with iron is indicated by the retention of high PFOR activity (Paunkov et al., 2022a). In the case of 638R^R^, the data do not allow a firm conclusion to be drawn because low intracellular iron levels brought about by reduced import of hemin, and therefore also of iron, can negatively affect both, FprA and cytochrome ubiquinol oxidase. It is important to note, however, that oxygen scavenging is totally abrogated when growth media are not supplemented with hemin (Paunkov et al., 2022b). Thus, oxygen scavenging is specifically retained in 638R^R^, albeit at a slower rate, whereas other enzyme activities such as PFOR are also lost as in hemin-deprived 638R (Paunkov et al., 2022a).

**Table 5:** Oxygen scavenging rates in *Bacteroides fragilis* strains.

Strain	Oxygen removed from medium after 1 h
638R*	53 ± 3%
638R^R^	37 ± 4%
638R nimA**	62 ± 2%
638R *nimA*^R^	33 ± 5%

*marks 638R > 638R^R^ *p* < 0.05, **marks 638R *nimA* > 638R *nimA*^R^ *p* < 0.05 according to Student’s *t*-test. All measurements were performed in three independent experiments each.

### 3.4. Metronidazole resistance can be widely abrogated in highly metronidazole-resistant 638R through addition of high concentrations of iron

**Figure 3:**
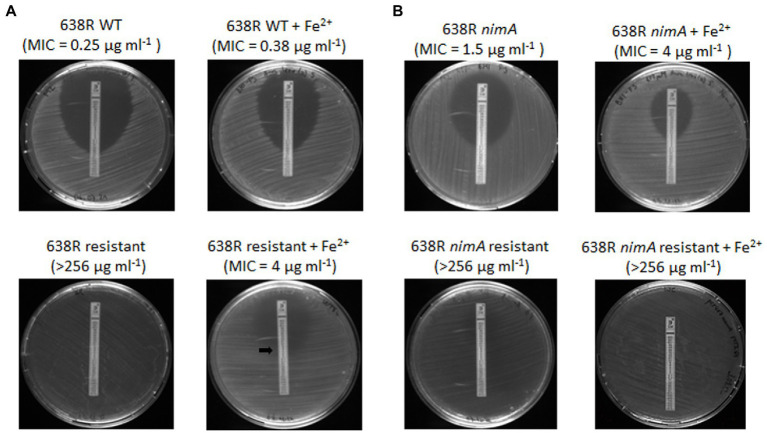
Susceptibilities of **(A)** 638R and 638R^R^, and **(B)** 638R *nimA* and 638R *nimA*^R^ as determined by Etest, either with or without supplementation of 100 μM ferrous iron sulfate. Due to the fainter appearance of 638R^R^ as compared to 638R, the point of intersection with the Etest strip is indicated by a black arrow.

Based on the results of the proteomic analysis of 638R^R^, we argued that metronidazole resistance could be caused by three different strategies: (1) inactivation of enzymes which depend on iron and/or heme for the reduction of metronidazole by depleting intracellular iron pools; (2) upregulation of RND transporters which pump metronidazole out of the cell; and (3) overexpression of ribosomal proteins and other proteins involved in protein synthesis which increases the tolerance to metronidazole by enabling the quick replacement of proteins damaged by reactive metronidazole intermediates (Leitsch, 2019). In order to assess the relative contribution of these three potential strategies, we exposed 638R^R^ to an iron source alternative to hemin, i.e., 100 μM ferrous iron sulfate, for one subculture in order to replenish the cells with iron. Ferrous iron sulfate had been described before to abrogate metronidazole resistance in a *feoAB* mutant (Veeranagouda et al., 2014) which is defective in importing ferrous iron released from heme (Rocha et al., 2019). After exposure to ferrous iron sulfate for one subculture, MICs to metronidazole were determined by Etest, likewise on plates with added 100 μM ferrous sulfate. Importantly, 638R^R^ did not show any inhibition by metronidazole according to Etest when plates were not supplemented with ferrous iron (Figure 3). However, when grown with ferrous iron sulfate, 638R^R^ displayed an MIC of 4 μg mL^−1^ (Figure 3A). This result indicates that the depletion of intracellular iron levels is the major strategy to achieve high-level metronidazole resistance in 638R and that other changes observed in the proteome of 638R^R^ confer protection against metronidazole to a smaller degree only. In contrast, the addition of 100 μM ferrous iron sulfate did only slightly if at all reduce the level of resistance in 638R *nimA*^R^ (Figure 3B). Further, the addition of ferrous iron sulfate had practically no effect on the susceptibility to metronidazole in 638R (Figure 3A). In 638R *nimA*, the addition of 100 μM ferrous iron even enhanced protection against metronidazole (Figure 3B) about twofold.

## 4. Discussion

Metronidazole resistance is a complex phenomenon in most anaerobes (reviewed in Leitsch, 2019), which is also underscored by the numerous changes in the proteome of *B*. *fragilis* 638R^R^ in this study. Our data suggest that the impairment of hemin import, and therefore also of iron import in general, is mainly responsible for the development of metronidazole resistance *in vitro* in the absence of the *nimA* gene. Although a large subset of proteins is differentially expressed in 638R^R^, supplementation with high concentrations of ferrous iron widely abrogates metronidazole resistance. This result mirrors observations by others (Veeranagouda et al., 2014) that metronidazole resistance as caused by impeded iron uptake *via* heme can be widely abolished through the addition of ferrous iron. The low-level protection against metronidazole remaining after the addition of ferrous iron as seen herein might be due to other changes such as the upregulation of RND transporters and of ribosomal proteins.

**Figure 4:**
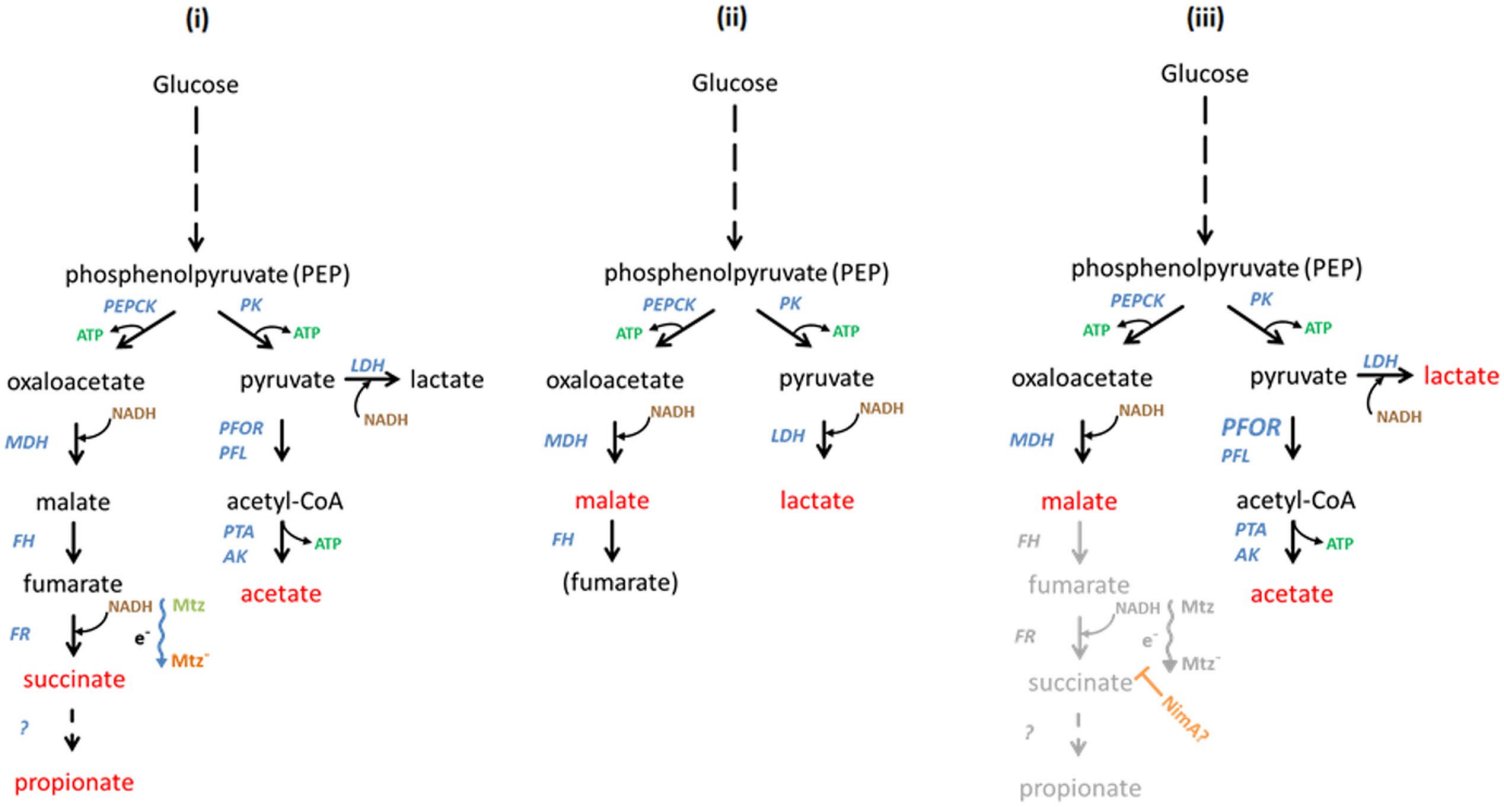
Hypothetical scheme of the interdependence of *Bacteroides fragilis* metabolism, induced metronidazole resistance, and of NimA-mediated protection from metronidazole, respectively. The main metabolic end products are indicated in red, the enzymes involved in blue, ATP in green, and NADH in brown. In the left panel **(i)** the pathways in *B*. *fragilis* leading to the formation of the metabolic end products (Frantz and McCallum, 1979) are depicted. Glucose is broken down to phosphoenolpyruvate (PEP) which can be either converted to oxaloacetate by phosphoenolpyruvate carboxykinase (PEPCK) or to pyruvate by pyruvate kinase (PK). Oxaloacetate is reduced to malate by malate dehydrogenase (MDH). Fumarate hydratase (FH), alternatively termed fumarase, dehydrates malate to fumarate which is then converted by fumarate reductase (FR) to succinate. Finally, succinate is converted to propionate by as yet unknown enzymes. The conversion of fumarate to succinate depends on an electron chain (indicated by e^−^ and a wavy arrow) which might be involved in metronidazole reduction (Mtz to Mtz^−^). Importantly, also cytochrome ubiquinol oxidase depends on this electron chain. Pyruvate is converted to acetyl-CoA by pyruvate:ferredoxin oxidoreductase (PFOR) and/or pyruvate formate-lyase (PFL). Ultimately, acetyl-CoA is converted to acetate by phosphotransacetylase (PTA) and acetate kinase (AK). Alternatively, pyruvate can be reduced to lactate by lactate dehydrogenase (LDH). Lactate, however, is not a major end product under normal conditions. In the middle panel **(ii)** the altered metabolism of 638R^R^ is shown. Due to the lack of iron and/or heme, PFOR and FR are not functional. Further, PFL is strongly downregulated. Consequently, NAD+ must be recycled through the reduction of pyruvate and oxaloacetate, rendering lactate and malate the major metabolic end products. Pathways for the reduction of metronidazole are shut-down. The loss of cytochrome ubiquinol oxidase activity is compensated by upregulation of FprA. The presumptive metabolism of 638R *nimA* is shown in the right panel **(iii)**. The formation of acetate is up due to increased activity of PFOR (indicated by enlarged letters). This is might be necessitated by a hypothetical interference of NimA (orange) with the electron chain which involved in the formation of succinate and could also lead to less metronidazole being reduced (inhibited pathway shown in gray). Importantly, this would also lead to a decreased activity of cytochrome ubiquinol oxidase, necessitating a compensatory upregulation of FprA. Finally, in order to ensure efficient regeneration of NAD+, more lactate could be formed in order to compensate for the decreased formation of succinate. Also malate might accumulate when formation of succinate is down.

The depletion of heme and iron has severe consequences for the activities of iron and heme-dependent enzymes which, consequently, cannot be equipped anymore with functional cofactors. We assume that the enzymes involved in energy metabolism which were found upregulated in this study, e.g., fumarate reductase or NADH:ubiquinone reductase are upregulated in the—probably futile—attempt to compensate for the loss of activity of iron and heme-dependent enzymes. This response, however, is not stereotypical because PFOR expression was not found altered in 638R^R^ although PFOR activity is lost early during the development of resistance (Narikawa et al., 1991; Paunkov et al., 2022a). Moreover, PFOR levels are also unaltered in 638R *nimA* although this strain displays a more than twofold higher PFOR activity as compared to 638R (Paunkov et al., 2022a). These observations indicate that gene expression studies alone are insufficient to resolve the mechanism behind metronidazole resistance. However, when combining the findings of our present study, of our previous study (Paunkov et al., 2022a), and of earlier studies on the effect of heme deprivation on metabolic end products in *B*. *fragilis* (Macy et al., 1975; Chen and Wolin, 1981), a scenario of the energy metabolism in 638R^R^ can be proposed (Figure 4). Three major end metabolites of the *B*. *fragilis* metabolism (Frantz and McCallum, 1979) are likely to be formed at much lower quantities or not at all: acetate, succinate, and propionate. Acetate is formed from acetyl-CoA, previously generated by PFOR and pyruvate formate-lyase (PFL), but PFOR is inactive due to the lack of iron–sulfur clusters which function as prosthetic groups, and PFL is downregulated approximately 11-fold (Table 4). Succinate is normally formed from fumarate by fumarate reductase and partly further processed to propionate, but fumarate reductase is dependent on the heme cofactor which is in short supply due to impaired heme import, resulting in strongly diminished activity (Paunkov et al., 2022a). Thus, the only major fermentative options remaining for the quantitative restoration of NAD+ are the reduction of pyruvate to lactate by lactate dehydrogenase (LDH) and the reduction of oxaloacetate to malate by malate dehydrogenase (MDH), resulting in far less ATP generated per mol of glucose (Figure 4). This is consistent with the impaired growth observed in 638R^R^ (Paunkov et al., 2022a), indicating that these changes come at a very high cost and are probably only tolerable under the optimal growth conditions of *in vitro* cell culture. In return, the decreased activities of iron-dependent enzymes or factors can be expected to confer resistance to metronidazole, arguably by slowing down metronidazole reduction, i.e., activation.

In 638R *nimA,* only five changes in the proteome were consistently observed as compared to 638R, one of them being the upregulation of flavodiiron protein FprA. Interestingly, the higher expression of FprA is a trait shared with 638R^R^, although it cannot be ruled out at present that in the latter this change is an attempt to compensate for low iron levels and does not lead to more actual FprA activity. Further, the induction of high-level resistance resulted in fewer changes in 638R *nimA* than in 638R and the significance of these changes is presently unclear. For financial reasons, the comparative analysis of 638R *nimA* and its resistant daughter strain was not repeated so it cannot be ruled out that many of the changes found would prove irreproducible in a second analysis, as was the case after repeating the analysis of 638R *nimA* vs. 638R. Still, the comparable paucity of changes in 638R *nimA*^R^ as compared to 638R^R^ (41 vs. 237) could be interpreted as compatible with the notion of Nim proteins acting as nitroreductases in the sense that direct detoxification of metronidazole by Nim would render extensive changes in protein expression unnecessary for survival. However, Nim levels are not further enhanced after induction of high-level resistance as has been repeatedly shown (Leitsch et al., 2014; Paunkov et al., 2022a; this study). This is hard to reconcile with a potential role of Nim proteins as nitroreductases which implies that more drug requires more enzyme to be metabolized. Further, said role would also not explain elevated PFOR activity as observed in 638R *nimA* and 638R *nimE* (Paunkov et al., 2022a). A clue to the solution of this conundrum might be the function of FprA. In *Bacteroides,* oxygen is reduced to water by cytochrome ubiquinol oxidase (Baughn and Malamy, 2004) and by FprA (Lu and Imlay, 2017). However, cytochrome ubiquinol oxidase is a heme-dependent enzyme and its function must be compromised in 638R^R^ which imports little to no hemin. A direct interaction of FprA with metronidazole is rather unlikely because overexpression of *fprA* in 638R did not confer any resistance to metronidazole, but upregulation of FprA might well be a strategy to compensate for impaired cytochrome ubiquinol oxidase activity. A potential interdependence of FprA overexpression and NimA is not obvious. However, the increased PFOR activity in 638R *nimA* (Paunkov et al., 2022a) indicates that metabolic flux might be redirected from succinate formation by fumarate reductase to acetate formation by the PFOR pathway (Figure 4). Fumarate reductase and cytochrome ubiquinol oxidase depend on electrons fueled into a quinone-based electron transport chain through the oxidation of NADH by NADH:ubiquinone reductase and NADH dehydrogenase (Ito et al., 2020). Any interference with this process would result in less reducing power being available for the formation of succinate by fumarate reductase and for the reduction of oxygen by cytochrome ubiquinol oxidase. An inhibition of cytochrome ubiquinol oxidase function, in turn, might necessitate the upregulation of FprA expression to ensure efficient oxygen scavenging. Importantly, this proposed interference might also have implications for metronidazole reduction. It is conceivable that leakage of electrons along the electron transport chain could lead to the formation of toxic metronidazole intermediates, and that less metronidazole would be reduced *via* this route if the flux of electrons was restricted. This might give other routes of reduction enough time to act, eventually resulting in the formation of less toxic products such as the corresponding aminoimidazole (Carlier et al., 1997). It is presently unclear how NimA could achieve this proposed interference mechanistically, but a metabolomic study targeting the metabolic end products in 638R with and without the *nimA* gene should provide an answer if our proposed model does indeed apply. If it applied, then 638R *nimA* should produce more acetate and malate and less succinate and propionate than 638R. This shift could even be more pronounced in 638R *nimA*^R^. Thus, further studies should focus on the concentrations of metabolic end products and of cofactors such as NAD+/NADH, NADP+/NADPH, FAD, FMN and quinones, in order to obtain a more complete picture of the effect of *nimA* or other *nim* genes on *B*. *fragilis* physiology. This would be a timely undertaking because metronidazole resistance in *Bacteroides* spp. is increasingly becoming a problem in clinical settings in certain countries (Vieira et al., 2006; Yehya et al., 2014; Sheikh et al., 2015).

## Data availability statement

The raw mass spectrometric data files presented in the study are deposited in the PRIDE repository, accession number PXD039809 (http://www.ebi.ac.uk/pride/archive/projects/PXD039809).

## Author contributions

AP performed the experiments, conceived the experiments, and analyzed the data. KH performed the mass-spectrometric analyses and analyzed the data. DS performed the experiments. JS conceived the experiments and analyzed the data. DL conceived the experiments, analyzed the data, and wrote the manuscript. All authors contributed to the article and approved the submitted version.

## Funding

This research was funded by the Austrian Science Fund (FWF) [grant number I 4234]. For the purpose of open access, the author has applied a CC BY public copyright license to any Author Accepted Manuscript version arising from this submission. Further, JS was funded by grant ANN_130760 from the National Research, Development and Innovation Office of Hungary (NKFIH).

## Conflict of interest

The authors declare that the research was conducted in the absence of any commercial or financial relationships that could be construed as a potential conflict of interest.

## Publisher’s note

All claims expressed in this article are solely those of the authors and do not necessarily represent those of their affiliated organizations, or those of the publisher, the editors and the reviewers. Any product that may be evaluated in this article, or claim that may be made by its manufacturer, is not guaranteed or endorsed by the publisher.

## Supplementary material

The Supplementary material for this article can be found online at: https://www.frontiersin.org/articles/10.3389/fmicb.2023.1158086/full#supplementary-material
