# Supplementary material for: Proteomic analysis of metronidazole resistance in the human facultative pathogen Bacteroides fragilis
Source: Front Microbiol. 2023 Mar 31;14:1158086. doi: 10.3389/fmicb.2023.1158086 (PMC10102347; doi:10.3389/fmicb.2023.1158086)
Supplement: Supplementary file 1 [file Table_1.DOCX]

**Supplementary Table 1**: Differentially expressed proteins in 638R *nimA* vs. 638R (first run).

All proteins at least up or down twofold. All proteins with p ≤ 0.01

| **Data base entry** | **Prot. ID**  **Analysis** | **Prot. ID**  **638R** | **Description** | **-fold up (+) or downregulation (-) in 638R *nimA*** |
| --- | --- | --- | --- | --- |
| BF638R_RS04550  BF638R_0963 | WP_005785280 | WP_005795977 | FprA family A-type flavoprotein [Bacteroides fragilis] | +3.7 |
| BF638R_RS04550  BF638R_0987 | WP_022347615 | WP_005795977 | bifunctional dihydroorotate dehydrogenase B NAD binding subunit/NADPH-dependent glutamate synthase [Bacteroides fragilis] | -2.3 |
| BF638R_RS05165  BF638R_1099 | WP_032556399 | WP_005785524 | HmuY family protein [Bacteroides fragilis] | +5.8 |
| BF638R_RS06280  BF638R_1339 | WP_005786073 | WP_005786073 | formate C-acetyltransferase [Bacteroides fragilis] | +4.1 |
| BF638R_RS07705  BF638R_1655 | WP_225549238 | WP_005786493 | 2-oxoacid:acceptor oxidoreductase family protein [Bacteroides fragilis] | -4.9 |
| BF638R_RS07740  BF638R_1663 | WP_005786505 | WP_005800633 | galactokinase [Bacteroides fragilis] | +8.6 |
| BF638R_RS08060  BF638R_1728 | WP_005794995 | WP_014298630 | exo-alpha-sialidase [Bacteroides fragilis] | -3.1 |
| BF638R_2202 | WP_130070829 | WP_014298858 | dihydrofolate reductase family protein [Bacteroides fragilis] | +2.0 |
| BF638R_RS10460  BF638R_2218 | WP_008660039 | WP_225550637 | hypothetical protein [Bacteroides fragilis] | +6.5 |
| BF638R_RS10470  BF638R_2220 | WP_005803364 | WP_005787354 | DUF4903 family protein [Bacteroides fragilis] | +5.6 |
| BF638R_RS12875  BF638R_2696 | WP_121972141 | WP_005793355 | flavodoxin FldA [Bacteroides fragilis] | +4.1 |
| BF638R_RS12910  BF638R_2704 | WP_005803797 | WP_005788333 | TonB-dependent receptor [Bacteroides fragilis] | +2.9 |
| BF638R_RS12945  BF638R_2714 | WP_005793337 | WP_005793337 | MAC/perforin domain-containing protein [Bacteroides fragilis] | +23.4 |
| BF638R_RS12950  BF638R_2715 | WP_005793335 | WP_005793335 | calycin-like domain-containing protein [Bacteroides fragilis] | +7.3 |
| BF638R_RS12975  BF638R_2721 | WP_005788367 | WP_005788367 | MotA/TolQ/ExbB proton channel family protein [Bacteroides fragilis] | +5.1 |
| BF638R_RS14455  BF638R_3025 | WP_195317159 | WP_005780311 | NADH peroxidase [Bacteroides fragilis] | -5.1 |
| BF638R_RS14595  BF638R_3053 | WP_032583887 | WP_005780364 | GGGtGRT protein, partial [Bacteroides fragilis] | -9.5 |
| BF638R_RS14745  BF638R_3087 | WP_032602110 | WP_005789204 | DUF4857 domain-containing protein [Bacteroides fragilis] | +2.1 |
| BF638R_RS14775  BF638R_3093 | WP_005798824 | WP_005789215 | DUF4876 domain-containing protein [Bacteroides fragilis] | +3.8 |
| BF638R_RS16620  BF638R_3466 | WP_005817293 | WP_014299312 | SusC/RagA family TonB-linked outer membrane protein [Bacteroides fragilis] | +8.6 |
| BF638R_RS16630  BF638R_3469 | WP_008769886 | WP_005817299 | insulinase family protein [Bacteroides fragilis] | +6.1 |
| BF638R_RS20210  BF638R_4171 | WP_005791441 | WP_014299549 | 1-deoxy-D-xylulose-5-phosphate synthase [Bacteroides fragilis] | +2.2 |
| BF638R_RS21465  BF638R_4436 | WP_196036602 | WP_009291521 | hypothetical protein [Bacteroides fragilis] | +9.5 |
|  | WP_172685633 |  | NimABCDEF family nitroimidazole resistance protein [Bacteroides fragilis] | +137.0 |

**Supplementary Table 2**: Differentially expressed proteins in 638R *nimA* (second run) as compared to 638R.

All proteins at least up or down twofold. All proteins with p ≤ 0.01

| **Data base entry** | **Prot. ID**  **Analysis** | **Prot. ID**  **638R** | **Description** | **-fold up (+) or downregulation (-) in 638R *nimA*** |
| --- | --- | --- | --- | --- |
| BF638R_RS00630  BF638R_0142 | WP_005783789 | WP_008657948 | TolC family protein [Bacteroides fragilis] | -2.3 |
| BF638R_RS02160  BF638R_0459 | WP_122130412 | WP_005784276 | glutamate decarboxylase [Bacteroides fragilis] | -2.4 |
| BF638R_RS02430  BF638R_0513 | WP_220391966 | WP_005784370 | outer membrane protein assembly factor BamA [Bacteroides fragilis] | -3 |
| BF638R_RS02575  BF638R_0540 | WP_032564179 | WP_005784415 | DUF4476 domain-containing protein [Bacteroides fragilis] | -3.5 |
| BF638R_RS02795  BF638R_0588 | WP_009291529 | WP_005784510 | NigD-like N-terminal domain-containing protein [Bacteroides fragilis] | -2 |
| BF638R_RS03050  BF638R_0639 | WP_115472011 | WP_005804383 | TonB-dependent receptor [Bacteroides fragilis] | -2.5 |
| BF638R_RS03425  BF638R_0716 | WP_220391818 | WP_014298245 | TonB-dependent receptor [Bacteroides fragilis] | -2 |
| BF638R_RS03710  BF638R_0777 | WP_005801381 | WP_014298260 | UpxZ family transcription anti-terminator antagonist [Bacteroides fragilis] | +2.1 |
| BF638R_RS03760  BF638R_0789 | WP_005784941 | WP_005784941 | polysaccharide biosynthesis protein [Bacteroides fragilis] | +2.6 |
| BF638R_RS03855  BF638R_0817 | WP_005787356 | WP_005787356 | TonB-dependent receptor [Bacteroides fragilis] | -2.4 |
| BF638R_RS04285  BF638R_0909 | WP_032532743 | WP_005785173 | aminoglycoside phosphotransferase family protein [Bacteroides fragilis] | -2.1 |
| BF638R_RS04355  BF638R_0924 | WP_005792451 | WP_014298310 | TonB-dependent receptor [Bacteroides fragilis] | -3.5 |
| BF638R_RS04550  BF638R_0963 | WP_005785280 | WP_005795977 | FprA family A-type flavoprotein [Bacteroides fragilis] | +12.9 |
| BF638R_RS05080  BF638R_1081 | WP_005795861 | WP_005795861 | UDP-N-acetyl-D-mannosamine dehydrogenase [Bacteroides fragilis] | +2 |
| BF638R_RS05085  BF638R_1082 | WP_005795861 | WP_005795861 | UDP-N-acetyl-D-mannosamine dehydrogenase [Bacteroides fragilis] | +2 |
| BF638R_RS05385  BF638R_1149 | WP_014298380 | WP_014298380 | restriction endonuclease subunit S [Bacteroides fragilis] | -4.7 |
| BF638R_RS06890  BF638R_1476 | WP_014298536 | WP_014298536 | porin [Bacteroides fragilis] | -4.2 |
| BF638R_RS07565  BF638R_1626 | WP_014298606 | WP_014298606 | acetyl-CoA carboxylase biotin carboxylase subunit [Bacteroides fragilis] | -2.3 |
| BF638R_RS07660  BF638R_1646 | WP_005795090 | WP_009291990 | perforin protein [Bacteroides fragilis] | -2.3 |
| BF638R_RS07740  BF638R_1663 | WP_005786505 | WP_005800633 | galactokinase [Bacteroides fragilis] | +12.5 |
| BF638R_RS08025  BF638R_1721 | WP_032576098 | WP_005795011 | SusD family outer membrane lipoprotein NanU [Bacteroides fragilis] | -7.6 |
| BF638R_RS08060  BF638R_1728 | WP_005794995 | WP_014298630 | exo-alpha-sialidase [Bacteroides fragilis] | -2.8 |
| BF638R_RS08435  BF638R_1803 | WP_254637929 | WP_004311273 | TonB-dependent receptor [Bacteroides fragilis] | -2.1 |
| BF638R_RS08960  BF638R_1909 | WP_032556626 | WP_005786918 | cytochrome ubiquinol oxidase subunit I [Bacteroides fragilis] | -4.4 |
| BF638R_RS09055  BF638R_1928 | WP_005800407 | WP_005786949 | SusC/RagA family TonB-linked outer membrane protein [Bacteroides fragilis] | -4 |
| BF9343_RS09570  BF9343_1960 | WP_005787151 | WP_005787151 | TonB-dependent receptor [Bacteroides fragilis] | -2.1 |
| BF638R_RS10400  BF638R_2202 | WP_014298858 | WP_014298858 | dihydrofolate reductase family protein [Bacteroides fragilis] | +2 |
| BF638R_RS10450  BF638R_2214 | WP_014298862 | WP_014298862 | PorT family protein [Bacteroides fragilis] | -2.2 |
| BF638R_RS10475  BF638R_2221 | WP_010992887 | WP_005787356 | TonB-dependent receptor [Bacteroides fragilis] | -2.4 |
| BF638R_RS12415  BF638R_2598  OR  BF638R_RS16720  BF638R_3486 | WP_005790531 | WP_005793443 | lipopolysaccharide biosynthesis protein RfbH [Bacteroides fragilis] | -3 |
| BF638R_RS12420  BF638R_2599 | WP_005803738 | WP_005788135 | UpxZ family transcription anti-terminator antagonist [Bacteroides fragilis] | -6.7 |
| BF638R_RS15345  BF638R_3206 | WP_225549466 | WP_014298405 | BT1926 family outer membrane beta-barrel protein [Bacteroides fragilis] | -2.1 |
| BF638R_RS15195  BF638R_3177 | WP_032573561 | WP_005789589 | TolC family protein [Bacteroides fragilis] | -2.4 |
| BF638R_RS16710  BF638R_3484 | WP_121963492 | WP_009292649 | CDP-glucose 4.6-dehydratase [Bacteroides fragilis] | -2.7 |
| BF638R_RS17380  BF638R_3622 | WP_005817459 | WP_014299354 | patatin-like phospholipase family protein [Bacteroides fragilis] | -3.1 |
| BF638R_RS17395  BF638R_3625 | WP_215710364 | WP_005781396 | NAD(P)-binding domain-containing protein. partial [Bacteroides fragilis] | -5 |
| BF638R_RS17495  BF638R_3645 | WP_014299369 | WP_014299369 | phage major capsid protein [Bacteroides fragilis] | +18.4 |
| BF638R_RS17780  BF638R_3701 | WP_032557366 | WP_005798215 | long-chain fatty acid--CoA ligase [Bacteroides fragilis] | -2.9 |
| BF638R_RS18230  BF638R_3792 | WP_032557366 | WP_005791103 | chorismate mutase, partial [Bacteroides fragilis]  bifunctional 3-deoxy-7-phosphoheptulonate synthase/chorismate mutase type II | -2.2 |
| BF638R_RS19770  BF638R_4078 | WP_005791482 | WP_005814336 | OprO/OprP family phosphate-selective porin [Bacteroides fragilis] | -2 |
| BF638R_RS20210  BF638R_4171 | WP_042987589 | WP_014299549 | 1-deoxy-D-xylulose-5-phosphate synthase [Bacteroides fragilis] | +2.4 |
| BF638R_RS20215  BF638R_4172 | WP_005791440 | WP_005791440 | flavodoxin [Bacteroides fragilis] | +2 |
| BF638R_4175 | WP_005797558 | WP_008661487 | hypothetical protein [Bacteroides fragilis] | -3,1 |
| BF638R_RS20575  BF638R_4248 | WP_005791297 | WP_014299580 | family 43 glycosylhydrolase [Bacteroides fragilis] | -2.6 |
| BF638R_RS21010  BF638R_4338 | WP_032557677 | WP_005791958 | TonB-dependent receptor [Bacteroides fragilis] | -2.6 |
| BF638R_RS21495  BF638R_4442 | WP_005805235 | WP_009291524 | RagB/SusD family nutrient uptake outer membrane protein [Bacteroides fragilis] | -2.2 |
|  | WP_172685633 |  | NimABCDEF family nitroimidazole resistance protein [Bacteroides fragilis] | +59.2 |

**Supplementary Table 3**: Differentially expressed proteins in 638R *nimA*^R^ as compared to 638R *nimA*.

| **Data base entry** | **ID**  **Analysis** | **ID**  **638R** | **Description** | **-fold up (+) or downregulation (-) in resistant 638R *nimA*** |
| --- | --- | --- | --- | --- |
| BF638R_RS02550  BF638R_0535 | WP_032577494 | WP_005784405 | amino acid permease [Bacteroides fragilis] | -16.7 |
| BF638R_RS03710  BF638R_0777 | WP_005801381 | WP_014298260 | UpxZ family transcription anti-terminator antagonist [Bacteroides fragilis] | +6.7 |
| BF638R_RS03720  BF638R_0779 | WP_032600526 | WP_005784921 | glucose-1-phosphate cytidylyltransferase [Bacteroides fragilis] | +3.6 |
| BF638R_RS03725  BF638R_0780 | WP_100766060 | WP_005784923 | CDP-glucose 4.6-dehydratase [Bacteroides fragilis] | +6.9 |
| BF638R_RS03730  BF638R_0781 | WP_005784923 | WP_005784923 | CDP-glucose 4.6-dehydratase [Bacteroides fragilis] | +6.9 |
| BF638R_RS03735  BF638R_0782 | WP_005784927 | WP_005784927 | Nucleoside-diphosphate-sugar epimerase (WcaG) | +7 |
| BF638R_RS03760  BF638R_0789 | WP_005784941 | WP_005784941 | NDP-sugar epimerase | +5.7 |
| BF638R_RS03765  BF638R_0790 | WP_014298264 | WP_014298264 | LegC family aminotransferase | +8.8 |
| BF638R_RS05050  BF638R_1075 | WP_005801178 | WP_014298343 | UpxZ family transcription anti-terminator antagonist [Bacteroides fragilis] | -2 |
| BF638R_RS05080  BF638R_1081 | WP_005795861 | WP_005795861 | UDP-N-acetyl-D-mannosamine dehydrogenase [Bacteroides fragilis] | -2.1 |
| BF638R_RS05100  BF638R_1085 | WP_005795850 | WP_005795850 | DegT/DnrJ/EryC1/StrS family aminotransferase [Bacteroides fragilis] | -3.4 |

All proteins at least up or down twofold. All proteins with p ≤ 0.01

| BF638R_RS06000  BF638R_1279 | WP_005785918 | WP_005816384 | DUF4890 domain-containing protein [Bacteroides fragilis] | -3.7 |
| --- | --- | --- | --- | --- |
| BF638R_RS06680  BF638R_1434 | WP_008768174 | WP_014298512 | UpxZ family transcription anti-terminator antagonist [Bacteroides fragilis] | -5.7 |
| BF638R_RS06700  BF638R_1438 | WP_014298515 | WP_014298515 | DegT/DnrJ/EryC1/StrS family aminotransferase [Bacteroides fragilis] | -4.1 |
| BF638R_RS06775  BF638R_1454 | WP_032586814 | WP_014298528 | glucose-1-phosphate thymidylyltransferase RfbA [Bacteroides fragilis] | -2.3 |
| BF638R_RS06795  BF638R_1458 | WP_008768196 | WP_014298531 | ketoacyl-ACP synthase III [Bacteroides fragilis] | -5.5 |
| BF638R_RS07205  BF638R_1544 | WP_032531450 | WP_005795239 | GNAT family N-acetyltransferase [Bacteroides fragilis] | -3.6 |
| BF638R_RS07225  BF638R_1548 | WP_022011156 | WP_014298569 | DegT/DnrJ/EryC1/StrS family aminotransferase [Bacteroides fragilis] | -2.3 |
| BF638R_RS07545  BF638R_1622 | WP_250717977 | WP_014298605 | L-lactate permease [Bacteroides fragilis] | -2.5 |
| BF638R_RS08730  BF638R_1863 | WP_005794845 | WP_005786815 | UpxZ family transcription anti-terminator antagonist [Bacteroides fragilis] | -3.3 |
| BF638R_RS08755  BF638R_1868 | WP_005817136 | WP_014298685 | phosphonopyruvate decarboxylase [Bacteroides fragilis] | -7 |
| BF638R_RS08760  BF638R_1869 | WP_014298686 | WP_014298686 | 2-aminoethylphosphonate--pyruvate transaminase [Bacteroides fragilis] | -5.6 |
| BF638R_RS08770  BF638R_1871 | WP_014298687 | WP_014298687 | DegT/DnrJ/EryC1/StrS family aminotransferase [Bacteroides fragilis] | -5 |
| BF638R_RS08810  BF638R_1879 | WP_005786856 | WP_014298695 | NDP-sugar epimerase | -4.1 |
| BF638R_RS09120  BF638R_1941 | WP_225481737 | WP_032577798 | hypothetical protein [Bacteroides fragilis] | -2.2 |
| BF638R_RS11550  BF638R_2435 | WP_005803571 | WP_005787831 | efflux RND transporter periplasmic adaptor subunit [Bacteroides fragilis] | -3.7 |
| BF638R_RS11675  BF638R_2458 | WP_041161475 | WP_041161475 | protein BatD [Bacteroides fragilis] | -2.4 |
| BF638R_RS12420  BF638R_2599 | WP_005803738 | WP_005788135 | UpxZ family transcription anti-terminator antagonist [Bacteroides fragilis] | +2.1 |
| BF638R_RS12515  BF638R_2619 | WP_005815482 | WP_005815482 | membrane protein insertase YidC [Bacteroides fragilis] | -5.6 |
| BF638R_RS13150  BF638R_2755 | WP_042985624 | WP_005788431 | V-type ATP synthase subunit D [Bacteroides fragilis] | -2.6 |
| BF638R_RS15080  BF638R_3153 | WP_032557143 | WP_005798883 | lamin tail domain-containing protein [Bacteroides fragilis]  new:  OadG family transporter subunit [Bacteroides fragilis] | -6 |
| BF638R_RS15185  BF638R_3174 | WP_022347707 | WP_005789585 | efflux RND transporter periplasmic adaptor subunit [Bacteroides fragilis] | -3.5 |
| BF638R_RS15225  BF638R_3183 | WP_005789602 | WP_005798918 | family 10 glycosylhydrolase [Bacteroides fragilis] | -3.1 |
| BF638R_RS16700  BF638R_3482 | WP_122288081 | WP_014299323 | GDP-mannose 4.6-dehydratase [Bacteroides fragilis] | -2 |
| BF638R_RS16730  BF638R_3488 | WP_011203398 | WP_005790533 | UpxZ family transcription anti-terminator antagonist [Bacteroides fragilis] | -3 |
| BF638R_RS17495  BF638R_3645 | WP_014299369 | WP_014299369 | phage major capsid protein [Bacteroides fragilis] | -14.6 |
| BF638R_RS17805  BF638R_3706 | WP_115474317 | WP_005790948 | MarR family transcriptional regulator [Bacteroides fragilis] | -4.7 |
| BF638R_RS18075  BF638R_3760 | WP_005791059 | WP_014299418 | Protoporphyrinogen oxidase HemY | -3.3 |
| BF638R_RS19420  BF638R_4011 | WP_005797639 | WP_005791581 | hypothetical protein [Bacteroides fragilis] | -2.2 |
| BF638R_RS19430  BF638R_4013 | WP_014299510 | WP_014299510 | 50S ribosomal protein L17 [Bacteroides fragilis] | -3.1 |
| BF638R_RS20390  BF638R_4206 | WP_032539182 | WP_005791373 | polyribonucleotide nucleotidyltransferase [Bacteroides fragilis] | -2.2 |

**Supplementary Table 4**: Differentially expressed proteins in 638R^R^ as compared to original 638R.

All proteins at least up or down twofold. All proteins with p ≤ 0.01

| **Data base entry** | **Prot. ID**  **Analysis** | **Prot. ID**  **638R** | **Description** | **-fold up (+) or downregulation (-) in resistant 638R *nimA*** |
| --- | --- | --- | --- | --- |
| BF638R_RS00100  BF638R_0020 | WP_032558772 | WP_005783570 | rubrerythrin family protein [Bacteroides fragilis] | +4.4 |
| BF638R_RS00125  BF638R_0025 | WP_005783582 | WP_005797086 | acetyl-CoA hydrolase/transferase family protein [Bacteroides fragilis] | +2 |
| BF638R_RS00315  BF638R_0070 | WP_010991895 | WP_011201856 | DUF4861 domain-containing protein [Bacteroides fragilis] | +23.2 |
| BF638R_RS00330  BF638R_0073 | WP_005797017 | WP_005783669 | DUF2723 domain-containing protein [Bacteroides fragilis] | +4.4 |
| F638R_RS00535  BF638R_0122 | WP_005813872 | WP_005783744 | tyrosine-protein phosphatase [Bacteroides fragilis] | +3.7 |
| BF638R_RS00570  BF638R_0129 | WP_005796876 | WP_008657933 | mannonate dehydratase [Bacteroides fragilis] | +2.8 |
| BF638R_RS00575  BF638R_0130 | WP_032577375 | WP_008657934 | SDR family oxidoreductase [Bacteroides fragilis] | +2.3 |
| BF638R_RS00720  BF638R_0160 | WP_005783820 | WP_008657970 | magnesium-translocating P-type ATPase [Bacteroides fragilis] | +8 |
| BF638R_RS00790  BF638R_0174 | WP_005801912 | WP_005783848 | glycosyltransferase family 8 protein [Bacteroides fragilis] | +3 |
| BF638R_RS00810  BF638R_0178 | WP_005813825 | WP_005796802 | ABC transporter ATP-binding protein [Bacteroides fragilis] | +11.5 |
| BF638R_RS00895  BF638R_0196 | WP_195341239 | WP_005801898 | hypothetical protein [Bacteroides fragilis] | +2.2 |
| BF638R_RS01515  BF638R_0326 | WP_022348188 | WP_005784029 | 1-acyl-sn-glycerol-3-phosphate acyltransferase [Bacteroides fragilis] | +4.9 |
| BF638R_RS01790  BF638R_0384 | WP_005784147 | WP_008658120 | DUF4982 domain-containing protein [Bacteroides fragilis] | +8.3 |
| BF638R_RS01845  BF638R_0395 | WP_005784174 | WP_008658132 | DUF3131 domain-containing protein [Bacteroides fragilis] | +3.5 |
| BF638R_RS02180  BF638R_0463 | WP_032560683 | WP_005779023 | TIGR00159 family protein [Bacteroides fragilis]  diadenylate cyclase CdaA | +5.8 |
| BF638R_RS02555  BF638R_0536 | WP_032587453 | WP_005784407 | two pore domain potassium channel family protein [Bacteroides fragilis] | +9.9 |
| BF638R_RS02635  BF638R_0554 | WP_005784439 | WP_005801569 | efflux RND transporter periplasmic adaptor subunit [Bacteroides fragilis] | +8.7 |
| BF638R_RS02660  BF638R_0559 | WP_250714269 | WP_005778830 | 50S ribosomal protein L19 [Bacteroides fragilis] | +5.7 |
| BF638R_RS02925  BF638R_0612 | WP_005784558 | WP_008658395 | mechanosensitive ion channel [Bacteroides fragilis] | +9.4 |
| BF638R_RS03020  BF638R_0633 | WP_057058491 | WP_005784668 | hypothetical protein [Bacteroides fragilis] | +4.8 |
| BF638R_RS03445  BF638R_0720 | WP_005801419 | WP_005784817 | hypothetical protein [Bacteroides fragilis] | +3.6 |
| BF638R_RS03485  BF638R_0728 | WP_005801413 | WP_014298250 | GH92 family glycosyl hydrolase [Bacteroides fragilis] | +6.3 |
| BF638R_RS03560  BF638R_0745 | WP_032556194 | WP_005784860 | ATP-dependent zinc metalloprotease FtsH [Bacteroides fragilis] | +6.3 |
| BF638R_RS03640  BF638R_0760 | WP_005784890 | WP_005796193 | DUF1573 domain-containing protein [Bacteroides fragilis] | +2.9 |
| BF638R_RS03715  BF638R_0778 | WP_005784919 | WP_005784919 | UDP-glucose dehydrogenase [Bacteroides fragilis] | -9 |
| BF638R_RS03725  BF638R_0780 | WP_005784923 | WP_005784923 | CDP-glucose 4.6-dehydratase [Bacteroides fragilis] | -5 |
| BF638R_RS03760  BF638R_0789 | WP_005784941 | WP_005784941 | NDP-sugar epimerase | -5.5 |
| BF638R_RS04175  BF638R_0886 | WP_032538477 | WP_005785120 | bifunctional UDP-3-O-[3-hydroxymyristoyl] N-acetylglucosamine deacetylase/3-hydroxyacyl-ACP dehydratase [Bacteroides fragilis] | +3.5 |
| BF638R_RS04210  BF638R_0893 | WP_014298297 | WP_014298297 | LemA family protein [Bacteroides fragilis] | +4.9 |
| BF638R_RS04250  BF638R_0901 | WP_032587802 | WP_005785160 | flotillin family protein [Bacteroides fragilis] | +7.3 |
| BF638R_RS04280  BF638R_0908 | WP_032532743 | WP_005785173 | aminoglycoside phosphotransferase family protein [Bacteroides fragilis] | +2.1 |
| BF638R_RS04440  BF638R_0940 | WP_005801257 | WP_014298316 | S41 family peptidase [Bacteroides fragilis] | +2.3 |
| BF638R_RS04470  BF638R_0946 | WP_121963858 | WP_005785250 | large-conductance mechanosensitive channel protein MscL [Bacteroides fragilis] | +6.4 |
| BF638R_RS04550  BF638R_0963 | WP_005785280 | WP_005795977 | FprA family A-type flavoprotein [Bacteroides fragilis] | +3.2 |
| BF638R_RS04560  BF638R_0965 | WP_005795974 | WP_005795974 | hypothetical protein [Bacteroides fragilis] | +3.1 |
| BF638R_RS04570  BF638R_0968 | WP_014298321 | WP_014298321 | AsmA family protein [Bacteroides fragilis] | +6.2 |
| BF638R_RS04600  BF638R_0974 | WP_005818611 | WP_005795956 | FecR domain-containing protein [Bacteroides fragilis] | +2.1 |
| BF638R_RS04625  BF638R_0979 | WP_005785313 | WP_005785313 | glycogen debranching enzyme N-terminal domain-containing protein [Bacteroides fragilis] | +2.9 |
| BF638R_RS04660  BF638R_0987 | WP_005785329 |  | bifunctional dihydroorotate dehydrogenase B NAD binding subunit/NADPH-dependent glutamate synthase [Bacteroides fragilis] | +2.3 |
| BF638R_RS04675  BF638R_0990 | WP_032532780 | WP_005785336 | 50S ribosomal protein L27 [Bacteroides fragilis] | +2.1 |
| BF638R_RS04715  BF638R_1001 | WP_010992246 | WP_010992246 | hypothetical protein [Bacteroides fragilis] | +4.1 |
| BF638R_RS04735  BF638R_1005 | WP_005785360 | WP_014298327 | DUF4836 family protein [Bacteroides fragilis] | +5.3 |
| BF638R_RS04760  BF638R_1010 | WP_223127444 | WP_014298329 | class I SAM-dependent rRNA methyltransferase [Bacteroides fragilis] | +2.8 |
| BF638R_RS04775  BF638R_1013 | WP_005785378 | WP_008768003 | DNA translocase FtsK [Bacteroides fragilis] | +9.3 |
| BF638R_RS04950  BF638R_1053 | WP_009291700 | WP_005785451 | membrane protein [Bacteroides fragilis] | +2.2 |
| BF638R_RS04985  BF638R_1061 | WP_220391015 | WP_005785463 | DUF4837 family protein [Bacteroides fragilis] | +2.3 |
| BF638R_RS05445  BF638R_1162 | WP_032570042 | WP_005795771 | SIR2 family protein. partial [Bacteroides fragilis] | +2.1 |
| BF638R_RS05040  BF638R_1072 | WP_014298342 | WP_014298342 | class I SAM-dependent methyltransferase [Bacteroides fragilis] | -8.4 |
| BF638R_RS05050  BF638R_1075 | WP_005801178 | WP_014298343 | UpxZ family transcription anti-terminator antagonist [Bacteroides fragilis] | -8.7 |
| BF638R_RS05080  BF638R_1081 | WP_005795861 | WP_005795861 | UDP-N-acetyl-D-mannosamine dehydrogenase [Bacteroides fragilis] | -7.9 |
| BF638R_RS05105  BF638R_1086 | WP_005795850 | WP_005795850 | DegT/DnrJ/EryC1/StrS family aminotransferase [Bacteroides fragilis] | -2.3 |
| BF638R_RS05165  BF638R_1099 | WP_032556399 | WP_005785524 | HmuY family protein [Bacteroides fragilis] | -509.4 |
| BF638R_RS05170  BF638R_1100 | WP_032564333 | WP_005793172 | TonB-dependent receptor [Bacteroides fragilis] | -77.2 |
| BF638R_RS05350  BF638R_1142 | WP_032572758 | WP_014298373 | type I restriction endonuclease subunit R [Bacteroides fragilis] | -18.4 |
| BF638R_RS05585  BF638R_1190 | WP_032572786 | WP_005785715 | flotillin-like protein FloA [Bacteroides fragilis] | +3.5 |
| BF638R_RS05690  BF638R_1211 | WP_081021470 | WP_005785759 | 50S ribosomal protein L25/general stress protein Ctc. partial [Bacteroides fragilis] | +4.5 |
| BF638R_RS05930  BF638R_1262 | WP_234077047 | WP_005785885 | catalase [Bacteroides fragilis] | +2.1 |
| BF638R_RS06000  BF638R_1279 | WP_005785918 | WP_005816384 | DUF4890 domain-containing protein [Bacteroides fragilis] | +11.5 |
| BF638R_RS06095  BF638R_1300 | WP_008768118 | WP_014298445 | OmpA family protein [Bacteroides fragilis] | +2.8 |
| BF638R_RS06100  BF638R_1302 | WP_011202355 | WP_008768120 | DUF488 family protein [Bacteroides fragilis] | +2.2 |
| BF638R_RS06230  BF638R_1329 | WP_005786050 | WP_014298454 | DUF1735 domain-containing protein [Bacteroides fragilis] | +14.1 |
| BF638R_RS06235  BF638R_1330 | WP_005786053 | WP_005800908 | DUF1735 domain-containing protein [Bacteroides fragilis] | +4.7 |
| BF638R_RS06275  BF638R_1338 | WP_032593684 | WP_008768141 | pyruvate formate lyase-activating protein [Bacteroides fragilis] | -10.8 |
| BF638R_RS06280  BF638R_1339 | WP_005786073 | WP_005786073 | formate C-acetyltransferase [Bacteroides fragilis] | -9.9 |
| BF638R_RS06650  BF638R_1427 | WP_032602326 | WP_032528770 | hypothetical protein [Bacteroides fragilis] | +10.7 |
| BF638R_RS06680  BF638R_1434 | WP_008768174 | WP_014298512 | UpxZ family transcription anti-terminator antagonist [Bacteroides fragilis] | -2.1 |
| BF638R_RS06740  BF638R_1446 | WP_014298522 | WP_014298522 | NTP transferase domain-containing protein [Bacteroides fragilis] | -3.9 |
| BF638R_RS07030  BF638R_1505 | WP_032562698 | WP_008768221 | helix-turn-helix domain-containing protein [Bacteroides fragilis] | +3.6 |
| BF638R_RS07145  BF638R_1531 | WP_005786308 | WP_014298556 | aspartate-alanine antiporter [Bacteroides fragilis] | +5.8 |
| BF638R_RS07205  BF638R_1544 | WP_032531450 | WP_005795239 | GNAT family N-acetyltransferase [Bacteroides fragilis] | -3.2 |
| BF638R_RS07305  BF638R_1564 | WP_005819214 | WP_005786352 | Rne/Rng family ribonuclease [Bacteroides fragilis] | +4 |
| BF638R_RS07310  BF638R_1566 | WP_049130181 | WP_005776335 | integration host factor subunit beta. partial [Bacteroides fragilis] | +3.8 |
| BF638R_RS07345  BF638R_1573 | WP_008768254 | WP_005786366 | (4Fe-4S)-binding protein [Bacteroides fragilis] | +2.4 |
| BF638R_RS07505  BF638R_1612 | WP_005786418 | WP_005786418 | NAD(P)/FAD-dependent oxidoreductase [Bacteroides fragilis] | +2.8 |
| BF638R_RS07565  BF638R_1626 | WP_014298606 | WP_014298606 | acetyl-CoA carboxylase biotin carboxylase subunit [Bacteroides fragilis] | +2.5 |
| BF638R_RS07635  BF638R_1641 | WP_005816898 | WP_014298612 | C10 family peptidase [Bacteroides fragilis] | +26.2 |
| BF638R_RS07650  BF638R_1644 | WP_005795094 | WP_005795094 | hypothetical protein [Bacteroides fragilis] | +2.4 |
| BF638R_RS07760  BF638R_1667 | WP_005800631 | WP_005800631 | hypothetical protein [Bacteroides fragilis] | +2.4 |
| BF638R_RS07770  BF638R_1669 | WP_175636904 | WP_005776445 | YtxH domain-containing protein [Bacteroides fragilis] | +5.8 |
| BF638R_RS07810  BF638R_1678 | WP_005786532 | WP_005786532 | adenylyl-sulfate kinase [Bacteroides fragilis] | +4.8 |
| BF638R_RS07840  BF638R_1684 | WP_042985292 | WP_032561129 | OmpA family protein [Bacteroides fragilis] | +4.1 |
| BF638R_RS07880  BF638R_1693 | WP_009292000 | WP_005786560 | OmpA family protein [Bacteroides fragilis] | +5.2 |
| BF638R_RS07910  BF638R_1699 | WP_032532337 | WP_008768315 | translation initiation factor IF-3 [Bacteroides fragilis] | +3.6 |
| BF638R_RS07915  BF638R_1700 | WP_084825280 | WP_005786574 | 50S ribosomal protein L35, partial [Bacteroides fragilis] | +7.3 |
| BF638R_RS07920  BF638R_1701 | WP_233483966 | WP_005786577 | 50S ribosomal protein L20 [Bacteroides fragilis] | +22.8 |
| BF638R_RS08025  BF638R_1721 | WP_032576098 | WP_005795011 | SusD family outer membrane lipoprotein NanU [Bacteroides fragilis] | +2.2 |
| BF638R_RS08060  BF638R_1728 | WP_005794995 | WP_014298630 | exo-alpha-sialidase [Bacteroides fragilis] | +2 |
| BF638R_RS08080  BF638R_1732 | WP_014298633 | WP_014298633 | glycoside hydrolase family 2 protein [Bacteroides fragilis] | -7.5 |
| BF638R_RS08085  BF638R_1733 | WP_032556518 | WP_008768326 | family 20 glycosylhydrolase [Bacteroides fragilis] | +7.2 |
| BF638R_RS08445  BF638R_1805 | WP_032539216 | WP_008768947 | hypothetical protein [Bacteroides fragilis] | -2.4 |
| BF638R_RS08450  BF638R_1806 | WP_032539215 | WP_005651733 | hypothetical protein [Bacteroides fragilis] | -2.9 |
| BF638R_RS08455  BF638R_1807 | WP_032539214 | WP_004311266 | HEAT repeat domain-containing protein [Bacteroides fragilis] | -14.2 |
| BF638R_RS08685  BF638R_1854 | WP_044820083 | WP_014298678 | hypothetical protein [Bacteroides fragilis] | +10.1 |
| BF638R_RS08730  BF638R_1863 | WP_005794845 | WP_005786815 | UpxZ family transcription anti-terminator antagonist [Bacteroides fragilis] | -6.2 |
| BF638R_RS08755  BF638R_1868 | WP_005817136 | WP_014298685 | phosphonopyruvate decarboxylase [Bacteroides fragilis] | -13.5 |
| BF638R_RS08765  BF638R_1870 | WP_014298687 | WP_014298687 | DegT/DnrJ/EryC1/StrS family aminotransferase [Bacteroides fragilis] | -8.4 |
| BF638R_RS08805  BF638R_1878 | WP_005786856 | WP_041161361 | UDP-N-acetylglucosamine 2-epimerase (non-hydrolyzing) [Bacteroides fragilis] | -3.8 |
| BF638R_RS08960  BF638R_1909 | WP_032556626 | WP_005786918 | cytochrome ubiquinol oxidase subunit I [Bacteroides fragilis] | +8.3 |
| BF638R_RS09035  BF638R_1925 | WP_005786942 | WP_005786942 | LemA family protein [Bacteroides fragilis] | +3.6 |
| BF638R_RS09120  BF638R_1941 | WP_225481737 | WP_032577798 | hypothetical protein [Bacteroides fragilis] | -22.7 |
| BF638R_RS09190  BF638R_1958 | WP_250717846 | WP_008768452 | OmpA family protein [Bacteroides fragilis] | +3 |
| BF638R_RS09590  BF638R_2038 | WP_195665805 | WP_014298755 | DUF3800 domain-containing protein [Bacteroides fragilis] | +6 |
| BF638R_RS10070  BF638R_2137 | WP_025814654 | WP_005787201 | NADH:ubiquinone reductase (Na(+)-transporting) subunit B [Bacteroides fragilis] | +8.5 |
| BF638R_RS10075  BF638R_2138 | WP_005787203 | WP_005787203 | Na(+)-translocating NADH-quinone reductase subunit C [Bacteroides fragilis] | +5.5 |
| BF638R_RS10090  BF638R_2141 | WP_005800144 | WP_005787210 | NADH:ubiquinone reductase (Na(+)-transporting) subunit F [Bacteroides fragilis] | +3.6 |
| BF638R_RS10170  BF638R_2157 | WP_005787240 | WP_005787240 | copper-translocating P-type ATPase [Bacteroides fragilis] | +3.1 |
| BF638R_RS10195  BF638R_2162 | WP_005817843 | WP_014298837 | oligopeptide transporter. OPT family [Bacteroides fragilis] | +8.7 |
| BF638R_RS10465  BF638R_2219 | WP_005787353 | WP_014298864 | hypothetical protein [Bacteroides fragilis] | -29.4 |
| BF638R_RS10470  BF638R_2220 | WP_005803364 | WP_005787354 | DUF4903 domain-containing protein [Bacteroides fragilis] | -130.2 |
| BF638R_RS10675  BF638R_2262 | WP_008768698 | WP_014298880 | hydroxylamine reductase [Bacteroides fragilis] | -2.6 |
| BF638R_RS10680  BF638R_2263 | WP_032562921 | WP_005787429 | Crp/Fnr family transcriptional regulator [Bacteroides fragilis] | +2.4 |
| BF638R_RS10770  BF638R_2281 | WP_005787468 | WP_005787468 | RelA/SpoT family protein [Bacteroides fragilis] | +4 |
| BF638R_RS10850  BF638R_2297 | WP_032529434 | WP_005803419 | efflux RND transporter periplasmic adaptor subunit [Bacteroides fragilis] | +3.8 |
| BF638R_RS10855  BF638R_2298 | WP_005787504 | WP_009292309 | efflux RND transporter permease subunit [Bacteroides fragilis] | +4.8 |
| BF638R_RS11550  BF638R_2435 | WP_005803571 | WP_005787831 | efflux RND transporter periplasmic adaptor subunit [Bacteroides fragilis] | +9.7 |
| BF638R_RS11555  BF638R_2436 | WP_032544118 | WP_014298941 | efflux RND transporter permease subunit [Bacteroides fragilis] | +8.6 |
| BF638R_RS11580  BF638R_2441 | WP_005787857 | WP_009292380 | dihydroorotate dehydrogenase electron transfer subunit [Bacteroides fragilis] | +2.9 |
| BF638R_RS11675  BF638R_2458 | WP_041161475 | WP_041161475 | protein BatD [Bacteroides fragilis]  Note „aerotolerance-related protein | +4.2 |
| BF638R_RS11690  BF638R_2461 | WP_146332951 | WP_005787921 | VWA domain-containing protein [Bacteroides fragilis] | +3.3 |
| BF638R_RS11730  BF638R_2470 | WP_049129612 | WP_002560155 | 50S ribosomal protein L33 [Bacteroides fragilis] | +5.9 |
| BF638R_RS11735  BF638R_2471 | WP_042985517 | WP_005787937 | 50S ribosomal protein L28 [Bacteroides fragilis] | +4.6 |
| BF638R_RS12065  BF638R_2534 | WP_005793529 | WP_041161478 | ATP-binding protein [Bacteroides fragilis] | +7.5 |
| BF638R_RS12250  BF638R_2565 | WP_032595946 | WP_014298999 | pyruvate. phosphate dikinase [Bacteroides fragilis] | +2.1 |
| BF638R_2567 | WP_230848698 | WP_008768963 | RluA family pseudouridine synthase [Bacteroides fragilis] | +26.1 |
| BF638R_RS12395  BF638R_2594 | WP_005793447 | WP_008657400 | thiamine pyrophosphate-binding protein [Bacteroides fragilis] | +2 |
| BF638R_RS12400  BF638R_2595 | WP_014299015 | WP_014299015 | CDP-glucose 4,6-dehydratase [Bacteroides fragilis] | +2.5 |
| BF638R_RS12415  BF638R_2598 | WP_053874081 | WP_005793443 | lipopolysaccharide biosynthesis protein RfbH [Bacteroides fragilis] | +2.4 |
| BF638R_RS12485  BF638R_2612 | WP_032590223 | WP_005788158 | electron transport complex subunit RsxC [Bacteroides fragilis] | +8.4 |
| BF638R_RS12525  BF638R_2621 | WP_01429902 | WP_014299021 | S9 family peptidase [Bacteroides fragilis] | +3.3 |
| BF638R_RS12530  BF638R_2622 | WP_005788177 | WP_005788177 | MBL fold metallo-hydrolase [Bacteroides fragilis] | +15 |
| BF638R_RS12870  BF638R_2695 | WP_005820599 | WP_005788311 | phosphoribosylaminoimidazolecarboxamide formyltransferase [Bacteroides fragilis] | -7.5 |
| BF638R_RS12895  BF638R_2701 | WP_032570843 | WP_005788325 | thioredoxin [Bacteroides fragilis]  = „TrxC“ (Paunkov et al. 2022) | -7.1 |
| BF638R_RS12905  BF638R_2703 | WP_005815412 | WP_005788329 | methionine synthase [Bacteroides fragilis] | -10.3 |
| BF638R_RS12945  BF638R_2714 | WP_005793337 | WP_005793337 | MAC/perforin domain-containing protein [Bacteroides fragilis] | -4.3 |
| BF638R_RS12950  BF638R_2715 | WP_005793335 | WP_005793335 | calycin-like domain-containing protein [Bacteroides fragilis] | -78.6 |
| BF638R_RS12955  BF638R_2716 | WP_010993105 | WP_010993105 | HmuY family protein [Bacteroides fragilis] | -19.9 |
| BF638R_RS12960  BF638R_2717 | WP_005788361 | WP_008660619 | TonB-dependent receptor [Bacteroides fragilis] | -10.8 |
| BF638R_RS12975  BF638R_2720 | WP_005788367 | WP_005788367 | MotA/TolQ/ExbB proton channel family protein [Bacteroides fragilis] | -53.4 |
| BF638R_RS13145  BF638R_2754 | WP_042985624 | WP_005788431 | V-type ATP synthase subunit D [Bacteroides fragilis] | +2.3 |
| BF638R_RS13145  BF638R_2754 | WP_005815364 | WP_008660668 | V-type ATP synthase subunit I [Bacteroides fragilis] | +4.6 |
| BF638R_RS12535  BF638R_2623 | WP_014299022 | WP_014299022 | PspC domain-containing protein [Bacteroides fragilis] | +2.4 |
| BF638R_RS14130  BF638R_2956 | WP_022348261 | WP_005788933 | hypothetical protein [Bacteroides fragilis] | +2.1 |
| BF638R_RS14315  BF638R_2994 | WP_005802959 | WP_005789010 | TolC family protein [Bacteroides fragilis] | +4.7 |
| BF638R_RS14505  BF638R_3035 | WP_005789128 | WP_005802910 | histidinol dehydrogenase [Bacteroides fragilis] | -2.4 |
| BF638R_RS14520  BF638R_3038 | WP_014299166 | WP_014299166 | PaaI family thioesterase [Bacteroides fragilis] | +2.5 |
| BF638R_RS14530  BF638R_3040 | WP_005802906 | WP_008661056 | thioredoxin family protein [Bacteroides fragilis] | +3.3 |
| BF638R_RS14595  BF638R_3053 | WP_032583887 | WP_005780364 | GGGtGRT protein, partial [Bacteroides fragilis] | -9.7 |
| BF638R_RS14645  BF638R_3066 | WP_014299181 | WP_014299181 | serine protease [Bacteroides fragilis] | +3.3 |
| BF638R_RS14745  BF638R_3087 | WP_032602110 | WP_005789204 | DUF4857 domain-containing protein [Bacteroides fragilis] | -103 |
| BF638R_RS14765  BF638R_3091 | WP_025812934 | WP_005789211 | hypothetical protein [Bacteroides fragilis] | -14 |
| BF638R_RS14775  BF638R_3093 | WP_005815019 | WP_005789215 | DUF4876 domain-containing protein [Bacteroides fragilis] | -26 |
| BF638R_RS14820  BF638R_3103 | WP_032563194 | WP_005789239 | OmpH family outer membrane protein [Bacteroides fragilis] | +6 |
| BF638R_RS14875  BF638R_3114 | WP_010993258 | WP_005789263 | beta-N-acetylhexosaminidase [Bacteroides fragilis] | +7.4 |
| BF638R_RS14935  BF638R_3126 | WP_014299208 | WP_014299208 | type I pullulanase [Bacteroides fragilis] | +5 |
| BF638R_RS15080  BF638R_3153 | WP_032557143 | WP_005798883. | lamin tail domain-containing protein [Bacteroides fragilis] | +19.1 |
| BF638R_RS15135  BF638R_3164 | WP_069187641 | WP_005789565 | type B 50S ribosomal protein L31, partial [Bacteroides fragilis] | +2.5 |
| BF638R_RS15165  BF638R_3170 | WP_115472655 | WP_005798907 | TonB-dependent receptor [Bacteroides fragilis] | +2.2 |
| BF638R_RS15185  BF638R_3174 | WP_022347707 | WP_005789585 | efflux RND transporter periplasmic adaptor subunit [Bacteroides fragilis] | +8.4 |
| BF638R_RS15190  BF638R_3176 | WP_005789587 | WP_008661179 | efflux RND transporter permease subunit [Bacteroides fragilis] | +2.1 |
| BF638R_RS15210  BF638R_3180 | WP_005789596 | WP_014299223 | response regulator [Bacteroides fragilis] | +8.1 |
| BF638R_RS15225  BF638R_3183 | WP_005789602 | WP_005798918 | family 10 glycosylhydrolase [Bacteroides fragilis] | +7.6 |
| BF638R_RS15270  BF638R_3191 | WP_011203237 | WP_005780701 | ATP-binding protein [Bacteroides fragilis] | +19.1 |
| BF638R_RS15330  BF638R_3203 | WP_005798942 | WP_005798942 | GH32 C-terminal domain-containing protein [Bacteroides fragilis] | +11.8 |
| BF638R_RS15400  BF638R_3217 | WP_005789670 | WP_005789670 | copper resistance protein NlpE [Bacteroides fragilis] | +8.1 |
| BF638R_RS15455  BF638R_3228 | WP_014299245 | WP_014299245 | alpha-L-fucosidase [Bacteroides fragilis] | -7.1 |
| BF638R_RS15780  BF638R_3297 | WP_032563370 | WP_005789838 | glycosyltransferase [Bacteroides fragilis] | +4.3 |
| BF638R_RS15855  BF638R_3306 | WP_005782543 | WP_008657121 | signal peptide peptidase SppA [Bacteroides fragilis] | +2.4 |
| BF638R_RS15890  BF638R_3312 | WP_005814768 | WP_008657154 | TetR/AcrR family transcriptional regulator [Bacteroides fragilis] | +2.1 |
| BF638R_RS16050  BF638R_3348 | WP_014299278 | WP_014299278 | hypothetical protein [Bacteroides fragilis] | +3.3 |
| BF638R_RS16335  BF638R_3408 | WP_032568403 | WP_005790373 | hypothetical protein [Bacteroides fragilis] | +10 |
| BF638R_RS16410  BF638R_3422 | WP_032528705 | WP_005790401 | LUD domain-containing protein [Bacteroides fragilis] | +5.1 |
| BF638R_RS16415  BF638R_3423 | WP_032528704 | WP_032528704 | (Fe-S)-binding protein [Bacteroides fragilis] | +3 |
| BF638R_RS16460  BF638R_3432 | WP_005781037 | WP_005790424 | ribonuclease Y [Bacteroides fragilis] | +4.6 |
| BF638R_RS16530  BF638R_3446 | WP_008769876 | WP_005790457 | hypothetical protein [Bacteroides fragilis] | -5.5 |
| BF638R_RS16615  BF638R_3465 | WP_005797886 | WP_014299311 | hypothetical protein [Bacteroides fragilis] | +3.8 |
| BF638R_RS16635  BF638R_3469 | WP_005790492 | WP_005817301 | ChaN family lipoprotein [Bacteroides fragilis] | -7.2 |
| BF638R_RS16655  BF638R_3473 | WP_005817308 | WP_014299316 | glucose-1-phosphate thymidylyltransferase RfbA [Bacteroides fragilis] | +4.7 |
| BF638R_RS16690  BF638R_3480 | WP_014299321 | WP_014299321 | glycosyltransferase [Bacteroides fragilis] | +5.8 |
| BF638R_RS16695  BF638R_3481 | WP_122288081 | WP_014299323 | GDP-mannose 4,6-dehydratase [Bacteroides fragilis] | +4 |
| BF638R_RS16720  BF638R_3486 | WP_005790531 | WP_009292648 | lipopolysaccharide biosynthesis protein RfbH [Bacteroides fragilis] | +2.2 |
| BF638R_RS16730  BF638R_3488 | WP_011203398 | WP_005790533 | UpxZ family transcription anti-terminator antagonist [Bacteroides fragilis] | +3.4 |
| BF638R_RS17040  BF638R_3553 | WP_146302179 | WP_005781259 | biopolymer transporter ExbD [Bacteroides fragilis] | +2.5 |
| BF638R_RS17050  BF638R_3555 | WP_005802355 | WP_005790652 | MotA/TolQ/ExbB proton channel family protein [Bacteroides fragilis] | +6.4 |
| BF638R_RS17695  BF638R_3685 | WP_005790911 | WP_014299391 | family 20 glycosylhydrolase [Bacteroides fragilis] | +2.5 |
| BF638R_RS17795  BF638R_3704 | WP_032559947 | WP_005790946 | 30S ribosomal protein S18, partial [Bacteroides fragilis] | +9.7 |
| BF638R_RS17805  BF638R_3706 | WP_115474317 | WP_005790948 | MarR family transcriptional regulator [Bacteroides fragilis] | +4.9 |
| BF638R_RS17815  BF638R_3709 | WP_057058610 | WP_005790953 | HAMP domain-containing histidine kinase [Bacteroides fragilis] | +14.3 |
| BF638R_RS17820  BF638R_3711 | WP_008769981 | WP_005790956 | elongation factor G [Bacteroides fragilis] | +6.8 |
| BF638R_RS17945  BF638R_3735 | WP_014299408 | WP_014299408 | [FeFe] hydrogenase H-cluster radical SAM maturase HydG [Bacteroides fragilis] | +3.2 |
| BF638R_RS18075  BF638R_3760 | WP_005791059 | WP_014299418 | Protoporphyrinogen oxidase HemY | -5.3 |
| BF638R_RS18125  BF638R_3770 | WP_005791076 | WP_005791076 | polysaccharide biosynthesis protein [Bacteroides fragilis] | -5.7 |
| BF638R_RS18465  BF638R_3833 | WP_005791704 | WP_014299443 | TolC family protein [Bacteroides fragilis] | -3.2 |
| BF638R_RS18545  BF638R_3849 | WP_225549554 | WP_014299450 | S8 family serine peptidase [Bacteroides fragilis] | -2.1 |
| BF638R_RS18605  BF638R_3862 | WP_005811166 | WP_005782434 | 50S ribosomal protein L13 [Bacteroides fragilis] | +7.6 |
| BF638R_RS18620  BF638R_3866 | WP_065344683 | WP_005791757 | pseudouridine synthase [Bacteroides fragilis] | +9.7 |
| BF638R_RS18675  BF638R_3874 | WP_005799114 | WP_005799114 | membrane protein [Bacteroides fragilis] | +.7 |
| BF638R_RS18685  BF638R_3876 | WP_250721834 | WP_005791774 | OmpA family protein [Bacteroides fragilis] | +3.5 |
| BF638R_RS18695  BF638R_3878 | WP_032576233 | WP_008661736 | FimB/Mfa2 family fimbrial subunit [Bacteroides fragilis] | +4.5 |
| BF638R_RS18710  BF638R_3882 | WP_032541896 | WP_005791781 | polymer-forming cytoskeletal protein [Bacteroides fragilis] | +5.9 |
| BF638R_RS18940  BF638R_3929 | WP_005814398 | WP_005814398 | peptidylprolyl isomerase [Bacteroides fragilis] | +8.5 |
| BF638R_RS19115  BF638R_3957 | WP_193684368 | WP_014299491 | M13 family metallopeptidase [Bacteroides fragilis] | +2.6 |
| BF638R_RS19360  BF638R_3999 | WP_005797651 | WP_014299503 | efflux RND transporter permease subunit [Bacteroides fragilis] | +5 |
| BF638R_RS19365  BF638R_4000 | WP_005791602 | WP_005791602 | efflux RND transporter periplasmic adaptor subunit [Bacteroides fragilis] | +9.5 |
| BF638R_RS19420  BF638R_4011 | WP_005797639 | WP_005791581 | hypothetical protein [Bacteroides fragilis] | +4.7 |
| BF638R_RS19430  BF638R_4013 | WP_014299510 | WP_014299510 | 50S ribosomal protein L17 [Bacteroides fragilis] | +8.9 |
| BF638R_RS19440  BF638R_4015 | WP_005791575 | WP_005791575 | 30S ribosomal protein S4, partial [Bacteroides fragilis] | +9.4 |
| BF638R_RS19450  BF638R_4017 | WP_235331997 | WP_002558050 | 30S ribosomal protein S13, partial [Bacteroides fragilis] | +8 |
| BF638R_RS19470  BF638R_4021 | WP_005791566 | WP_005804135 | 50S ribosomal protein L15 [Bacteroides fragilis] | +6.7 |
| BF638R_RS19490  BF638R_4025 | WP_049132868 | WP_005791561 | 50S ribosomal protein L6, partial [Bacteroides fragilis] | +6.8 |
| BF638R_RS19495  BF638R_4026 | WP_032584693 | WP_005782213 | 30S ribosomal protein S8. partial [Bacteroides fragilis] | +3.5 |
| BF638R_RS19500  BF638R_4027 | WP_005791558 | WP_005791558 | 30S ribosomal protein S14 [Bacteroides fragilis] | +8.2 |
| BF638R_RS19505  BF638R_4028 | WP_065850126 | WP_005791554 | 50S ribosomal protein L24 [Bacteroides fragilis] | +4.5 |
| BF638R_RS19510  BF638R_4029 | WP_005791556 | WP_005791556 | 50S ribosomal protein L5 [Bacteroides fragilis] | +3.3 |
| BF638R_RS19530  BF638R_4034 | WP_005811371 | WP_005791549 | 50S ribosomal protein L16 [Bacteroides fragilis] | +13.2 |
| BF638R_RS19540  BF638R_4036 | WP_230848688 | WP_004291236 | 50S ribosomal protein L22 [Bacteroides fragilis] | +9.9 |
| BF638R_RS19545  BF638R_4037 | WP_057059003.1 | WP_005782197 | 30S ribosomal protein S19, partial [Bacteroides fragilis] | +12.7 |
| BF638R_RS19550  BF638R_4038 | WP_225550726 | WP_005791545 | 50S ribosomal protein L2 [Bacteroides fragilis] | +12.8 |
| BF638R_RS19560  BF638R_4040 | WP_032574063 | WP_005782191 | 50S ribosomal protein L4 [Bacteroides fragilis] | +2.4 |
| BF638R_RS19570  BF638R_4042 | WP_057058737 | WP_005782187 | 30S ribosomal protein S10. partial [Bacteroides fragilis] | +3.5 |
| BF638R_RS19580  BF638R_4044 | WP_250746651 | WP_005791536 | 30S ribosomal protein S7 [Bacteroides fragilis] | +14.5 |
| BF638R_RS19595  BF638R_4047 | WP_005797631 | WP_005791531 | YtxH domain-containing protein [Bacteroides fragilis] | +10.4 |
| BF638R_RS19635  BF638R_4055 | WP_044821031 | WP_005782161 | 50S ribosomal protein L1 [Bacteroides fragilis] | +5.4 |
| BF638R_RS19695  BF638R_4063 | WP_155268837 | WP_005782152 | 30S ribosomal protein S21, partial [Bacteroides fragilis] | +2.2 |
| BF638R_RS20430  BF638R_4217 | WP_223127000 | WP_014299572 | butyrate kinase [Bacteroides fragilis] | +10.9 |
| BF638R_RS20450  BF638R_4221 | WP_005791343 | WP_005797516 | M28 family peptidase [Bacteroides fragilis] | +2.7 |
| BF638R_RS20490  BF638R_4229 | WP_005791327 | WP_014299574 | calcium-translocating P-type ATPase. PMCA-type [Bacteroides fragilis] | +3.9 |
| BF638R_RS20505  BF638R_4232 | WP_065344719 | WP_005791319 | Na/Pi cotransporter family protein [Bacteroides fragilis] | +5.2 |
| BF638R_RS20850  BF638R_4305 | WP_081021439 | WP_005791188 | HU family DNA-binding protein. partial [Bacteroides fragilis] | +2.8 |
| BF638R_RS20875  BF638R_4310 | WP_005797413 | WP_005791181 | protein translocase subunit SecDF [Bacteroides fragilis] | +6 |
| BF638R_RS21025  BF638R_4341 | WP_052574877 | WP_005791955 | 30S ribosomal protein S15, partial [Bacteroides fragilis] | +7.1 |
| BF638R_RS21135  BF638R_4366 | WP_005791935 | WP_008661629 | 6-bladed beta-propeller [Bacteroides fragilis] | +2.9 |
| BF638R_RS21330  BF638R_4407 | WP_175393078 | WP_025813753 | OmpA family protein [Bacteroides fragilis] | +5.9 |
| BF638R_RS21750  BF638R_4485 | WP_005822469 | WP_005814060 | DUF4469 domain-containing protein [Bacteroides fragilis] | +7.1 |
| BF638R_RS21815  BF638R_4499 | WP_195341926 | WP_005783488 | succinate dehydrogenase/fumarate reductase cytochrome b subunit [Bacteroides fragilis] | +11.5 |
| BF638R_RS21835  BF638R_4503 | WP_005783495 | WP_008657783 | DUF4374 domain-containing protein [Bacteroides fragilis] | -70.8 |
| BF638R_RS21840  BF638R_4504 | WP_009291280 | WP_005783497 | TonB-dependent receptor [Bacteroides fragilis] | -32.2 |

**Supplementary Table 5**: Differentially expressed proteins as shown in Supplementary Table 6 grouped into the following subsets defined by localization or function:

7.1 Surface proteins

7.2. Protein synthesis

7.3. DNA and RNA integrity

7.4. Transcriptional regulators

7.5. Antimicrobial resistance-related

7.6. Energy metabolism

7.7. (N-)Sugar metabolism

7.8. Peptidases

7.9. Other Proteins and enzymes

7.10. Hypothetical proteins and proteins with domains without known functions

**7.1 Surface proteins (84/237): Transporters/pores marked blue (41/84)**

| **Data base entry** | **description** | **Notes** | **-fold up (+) or downregulation (-) in resistant 638R *nimA*** |
| --- | --- | --- | --- |
| BF638R_RS00535  BF638R_0122 | tyrosine-protein phosphatase [Bacteroides fragilis] | The classical protein tyrosine phosphatases (PTPs) can be categorized as receptor-like (R) or non-transmembrane (NT) proteins. | +3.7 |
| BF638R_RS00590  BF638R_0133 | S26 family signal peptidase [Bacteroides fragilis] | Peptidases S26A (signal peptidase I) removes the hydrophobic, N-terminal signal peptides as proteins are translocated across membranes. | +10.8 |
| BF638R_RS00720  BF638R_0160 | magnesium-translocating P-type ATPase [Bacteroides fragilis] | MgtA is a membrane protein which actively transports Mg(2+) into the cytosol with its electro-chemical gradient rather than against the gradient as other cation transporters do. It may act both as a transporter and as a sensor for Mg(2+). In Salmonella typhimurium and Escherichia coli, the two-component system PhoQ/PhoP regulates the transcription of the mgtA gene by sensing Mg(2+) concentrations in the periplasm. MgtA is activated by cardiolipin and it highly sensitive to free magnesium in vitro. It consists of a transmembrane domain and three cytosolic domains: nucleotide-binding domain, phosphorylation domain and actuator domain, and belongs to the P-type ATPase type III subfamily. | +8 |
| BF638R_RS00810  BF638R_0178 | ABC transporter ATP-binding protein [Bacteroides fragilis] | MdlB | +11.5 |
| BF638R_RS02555  BF638R_0536 | two pore domain potassium channel family protein [Bacteroides fragilis] | The two-pore-domain or tandem pore domain potassium channels are a family of 15 members that form what is known as leak channels. Consist of four transmembrane segments | +9.9 |
| BF638R_RS02635  BF638R_0554 | efflux RND transporter periplasmic adaptor subunit [Bacteroides fragilis] | AcrA | +8.7 |
| BF638R_RS02925  BF638R_0612 | mechanosensitive ion channel [Bacteroides fragilis] | Mechanosensitive channels respond to membrane tension by altering their conformation between an open state and a closed state. | +9.4 |
| BF638R_RS03560  BF638R_0745 | ATP-dependent zinc metalloprotease FtsH [Bacteroides fragilis] | Acts as a processive, ATP-dependent zinc metallopeptidase for both cytoplasmic and membrane proteins. Plays a role in the quality control of integral membrane proteins. | +6.3 |
| BF638R_RS04210  BF638R_0893 | LemA family protein [Bacteroides fragilis] | The members of this family are related to the LemA protein Swiss:P71452 [PMID:8758895]. LemA contains an amino terminal predicted transmembrane helix. | +4.9 |
| BF638R_RS04250  BF638R_0901 | flotillin family protein [Bacteroides fragilis] | Bacterial flotillin-like proteins are found in membrane microdomains that may be equivalent to eukaryotic membrane rafts. | +7.3 |
| BF638R_RS04470  BF638R_0946 | large-conductance mechanosensitive channel protein MscL [Bacteroides fragilis] | The Large Conductance Mechanosensitive Ion Channel (MscL) Family consists of pore-forming membrane proteins that are responsible for translating physical forces applied to cell membranes into electrophysiological activities. Ion channel. | +6.4 |
| BF638R_RS04570  BF638R_0968 | AsmA family protein [Bacteroides fragilis] | Involved in the inhibition of assembly of mutant ompF proteins. In general, could be involved in the assembly of outer membrane proteins. | +5.6 |
| BF638R_RS04600  BF638R_0974 | FecR domain-containing protein [Bacteroides fragilis] | Regulation of iron dicitrate transport. In the absence of citrate FecR inactivates fecI. FecR is probably a sensor that recognizes iron dicitrate in the periplasm. | +4.8 |
| BF638R_RS04775  BF638R_1013 | DNA translocase FtsK [Bacteroides fragilis] | FtsK is a double-stranded DNA translocase, a motor that converts the chemical energy of binding and hydrolysing ATP into movement of a DNA substrate. Escherichia coli FtsK is a septum-located DNA translocase that co-ordinates the late stages of cytokinesis and chromosome segregation. | +9.3 |
| BF638R_RS04950  BF638R_1053 | membrane protein [Bacteroides fragilis] |  | +2.,2 |
| BF638R_RS05165  BF638R_1099 | HmuY family protein [Bacteroides fragilis] | HmuY is a novel heme-binding protein that recruits heme from host carriers and delivers it to its cognate outer-membrane transporter, the TonB-dependent receptor HmuR. This family of proteins is found in bacteria. Proteins in this family are typically between 214 and 278 amino acids in length. | -509.4 |
| BF638R_RS05170  BF638R_1100 | TonB-dependent receptor [Bacteroides fragilis] | Outer membrane receptors, also known as TonB-dependent receptors, are a family of beta barrel proteins named for their localization in the outer membrane of gram-negative bacteria. TonB complexes sense signals from the outside of bacterial cells and transmit them into the cytoplasm, leading to transcriptional activation of target genes. TonB-dependent receptors in gram-negative bacteria are associated with the uptake and transport of large substrates such as iron siderophore complexes and vitamin B12. | -77.2 |
| BF638R_RS05585  BF638R_1190 | flotillin-like protein FloA [Bacteroides fragilis] | Bacterial flotillin-like proteins are found in membrane microdomains that may be equivalent to eukaryotic membrane rafts. | +3.5 |
| BF638R_RS06095  BF638R_1300 | OmpA family protein [Bacteroides fragilis] | The OmpA domain is a conserved protein domain with a beta/alpha/beta/alpha-beta(2) structure found in the C-terminal region of many Gram-negative bacterial outer membrane proteins, such as porin-like integral membrane proteins (such as ompA), small lipid-anchored proteins (such as pal), and MotB proton channels. | +2.8 |
| BF638R_RS07145  BF638R_1531 | aspartate-alanine antiporter [Bacteroides fragilis] |  | +5.8 |
| BF638R_RS07840  BF638R_1684 | OmpA family protein [Bacteroides fragilis] |  | +4.1 |
| BF638R_RS07880  BF638R_1693 | OmpA family protein [Bacteroides fragilis] |  | +5.2 |
| BF638R_RS08025  BF638R_1721 | SusD family outer membrane lipoprotein NanU [Bacteroides fragilis] | NanU, related to SusD and RagB, is an outer membrane lipoprotein from a TonB-dependent nutrient uptake complex | +2.,2 |
| BF638R_RS08035  BF638R_1723 | TonB-dependent receptor [Bacteroides fragilis] |  | +3.9 |
| BF638R_RS08060  BF638R_1728 | exo-alpha-sialidase [Bacteroides fragilis] | Neuraminidase. Exo-α-sialidase is a glycoside hydrolase that cleaves the glycosidic linkages of neuraminic acids | +2 |
| BF638R_RS08960  BF638R_1909 | cytochrome ubiquinol oxidase subunit I [Bacteroides fragilis] | Cytochrome bd terminal oxidase subunit I | +8.3 |
| BF638R_RS09035  BF638R_1925 | LemA family protein [Bacteroides fragilis] |  | +3.6 |
| BF638R_RS09190  BF638R_1958 | OmpA family protein [Bacteroides fragilis] |  | +3 |
| BF638R_RS10070  BF638R_2137 | NADH:ubiquinone reductase (Na(+)-transporting) subunit B [Bacteroides fragilis] | The sodium -pumping NADH: ubiquinone oxidoreductase (Na+-NQR) is the main ion pump and the primary entry site for electrons into the respiratory chain of many different types of pathogenic bacteria. This enzymatic complex creates a transmembrane gradient of sodium that is used by the cell to sustain ionic homeostasis, nutrient transport, ATP synthesis, flagellum rotation and other essential processes. | +8.5 |
| BF638R_RS10075  BF638R_2138 | Na(+)-translocating NADH-quinone reductase subunit C [Bacteroides fragilis] | See above. | +5.5 |
| BF638R_RS10090  BF638R_2141 | NADH:ubiquinone reductase (Na(+)-transporting) subunit F [Bacteroides fragilis] | See above. | +3.6 |
| BF638R_RS10170  BF638R_2157 | copper-translocating P-type ATPase [Bacteroides fragilis] | These proteins catalyze ATP-dependent copper transport across cell membranes for the metallation of many essential cuproenzymes, as well as for the removal of excess cellular copper to prevent copper toxicity. | +3.,1 |
| BF638R_RS10195  BF638R_2162 | oligopeptide transporter. OPT family [Bacteroides fragilis] | The transporter OPT family are transporters of small oligopeptides | +8.7 |
| BF638R_RS10850  BF638R_2297 | efflux RND transporter periplasmic adaptor subunit [Bacteroides fragilis] | RND (Resistance-Nodulation-Division) family transporters are widespread especially among Gram-negative bacteria, and catalyze the active efflux of many antibiotics and chemotherapeutic agents. They have very large periplasmic domains, and form tripartite complexes with outer membrane channels and periplasmic adaptor proteins.  AcrA | +3.8 |
| BF638R_RS10855  BF638R_2298 | efflux RND transporter permease subunit [Bacteroides fragilis] | AcrB | +4.8 |
| BF638R_RS11550  BF638R_2435 | efflux RND transporter periplasmic adaptor subunit [Bacteroides fragilis] | AcrA | +9.7 |
| BF638R_RS11555  BF638R_2436 | efflux RND transporter permease subunit [Bacteroides fragilis] | AcrB | +8.6 |
| BF638R_RS11675  BF638R_2458 | protein BatD [Bacteroides fragilis] | This family of proteins carries up to three membrane spanning regions and is involved in tolerance to oxygen in in Bacteroides species. See Mol. Microbiol. 32, 139-49, (1999)  Bacteroides aerotolerance. Membrane protein. | +4.2 |
| BF638R_RS12415  BF638R_2598 | lipopolysaccharide biosynthesis protein RfbH [Bacteroides fragilis] | Bacterial outer membrane biogenesis; LPS O-antigen biosynthesis. | +2.4 |
| BF638R_RS12485  BF638R_2612 | electron transport complex subunit RsxC [Bacteroides fragilis] | Part of a membrane complex involved in electron transport. Required to maintain the reduced state of SoxR. | +8.4 |
| BF638R_RS12530  BF638R_2622 | MBL fold metallo-hydrolase [Bacteroides fragilis] | Members of the MBL-fold metallohydrolase superfamily are mainly hydrolytic enzymes which carry out a variety of biological functions. The class B metal beta-lactamases (MBLs) for which this fold was named perform only a small fraction of the activities included in this superfamily. | +15 |
| BF638R_RS12950  BF638R_2715 | calycin-like domain-containing protein [Bacteroides fragilis] | Calycins form a large protein superfamily that share similar β-barrel structures. Calycins can be divided into families that include lipocalins, fatty acid binding proteins, triabin, and thrombin inhibitor | -78.6 |
| BF638R_RS12955  BF638R_2716 | HmuY family protein [Bacteroides fragilis] | HmuY is a novel heme-binding protein that recruits heme from host carriers and delivers it to its cognate outer-membrane transporter, the TonB-dependent receptor HmuR. This family of proteins is found in bacteria. Proteins in this family are typically between 214 and 278 amino acids in length. | -19.9 |
| BF638R_RS12960  BF638R_2717 | TonB-dependent receptor [Bacteroides fragilis] |  | -10.8 |
| BF638R_RS12975  BF638R_2720 | MotA/TolQ/ExbB proton channel family protein [Bacteroides fragilis] |  | -53.4 |
| BF638R_RS13145  BF638R_2754 | V-type ATP synthase subunit I [Bacteroides fragilis] | Transmembrane ATPases are membrane-bound enzyme complexes/ion transporters that use ATP hydrolysis to drive the transport of protons across a membrane. | +4.6 |
| BF638R_RS12535  BF638R_2623 | PspC domain-containing protein [Bacteroides fragilis] | PspC is a multifunctional surface-exposed choline-binding protein displaying various adhesive properties.  phage shock protein C | +2.4 |
| BF638R_RS14315  BF638R_2994 | TolC family protein [Bacteroides fragilis] | TolC and its homologues are involved in the export of chemically diverse molecules ranging from large protein toxins, such as alpha-hemolysin, to small toxic compounds, such as antibiotics. TolC family members thus play important roles in conferring pathogenic bacteria with both virulence and multidrug resistance. | +4.7 |
| BF638R_RS14530  BF638R_3040 | thioredoxin family protein [Bacteroides fragilis] --> DsbD | DsbD is a unique three-domain protein that controls the redox state of the periplasm by transferring reductant from cytoplasmic thioredoxin via a thiol:disulfide cascade. DsbD is essential for cytochrome c maturation and much progress has been made in understanding how it functions | +3.3 |
| BF638R_RS14820  BF638R_3103 | OmpH family outer membrane protein [Bacteroides fragilis] | OmpH has been characterised as a molecular chaperone that interacts with unfolded proteins as they emerge in the periplasm from the Sec translocation machinery | +6.2 |
| BF638R_RS14875  BF638R_3114 | beta-N-acetylhexosaminidase [Bacteroides fragilis] | n general, hexosaminidases describe enzymes that cleave the glycosidic linkage of 2-acetamido-2-deoxy-β-d-glycosides. This group of enzymes includes, for example, lysozymes, chitinases, chitobioses, hyaluronidases, O-GlcNAcase, and others. | +7.4 |
| BF638R_RS14935  BF638R_3126 | type I pullulanase [Bacteroides fragilis] | Pullulanase is a specific kind of glucanase, an amylolytic exoenzyme, that degrades pullulan. | +5 |
| BF638R_RS15080  BF638R_3153 | lamin tail domain-containing protein [Bacteroides fragilis] | Likely a membrane protein. | +19.1 |
| BF638R_RS15165  BF638R_3170 | TonB-dependent receptor [Bacteroides fragilis] |  | +2.,2 |
| BF638R_RS15185  BF638R_3174 | efflux RND transporter periplasmic adaptor subunit [Bacteroides fragilis] | AcrA | +8.4 |
| BF638R_RS15190  BF638R_3176 | efflux RND transporter permease subunit [Bacteroides fragilis] | AcrB | +2.1 |
| BF638R_RS15210  BF638R_3180 | response regulator [Bacteroides fragilis] | Surface-associated supercomplex signal transducer! | +8.1 |
| BF638R_RS15400  BF638R_3217 | copper resistance protein NlpE [Bacteroides fragilis] | This family represents a bacterial outer membrane lipoprotein that is necessary for signalling by the Cpx pathway. This pathway responds to cell envelope disturbances and increases the expression of periplasmic protein folding and degradation factors. | +8.1 |
| BF638R_RS15780  BF638R_3297 | glycosyltransferase [Bacteroides fragilis] | WcaA-like. Glycosyltransferase involved in cell wall bisynthesis | +4.3 |
| BF638R_RS15855  BF638R_3306 | signal peptide peptidase SppA [Bacteroides fragilis] | Signal peptide peptidase (SPP) is a membrane-bound enzyme that uses a serine/lysine catalytic dyad mechanism to cleave the remnant signal peptides in the cellular membrane and aids in protein secretion. | +2.4 |
| BF638R_RS16635  BF638R_3469 | ChaN family lipoprotein [Bacteroides fragilis] | A domain found in ChaN, a heme-binding/iron-regulated lipoprotein from Campylobacter jejuni. ChaN, possibly involved in the uptake of heme-iron, contains a pair of cofacial heme groups situated between two ChaN monomers.  Haem-binding uptake, Tiki superfamily, ChaN | -7.2 |
| BF638R_RS16690  BF638R_3480 | glycosyltransferase [Bacteroides fragilis] | WcaA-like. Glycosyltransferase involved in cell wall bisynthesis. | +5.8 |
| BF638R_RS16720  BF638R_3486 | lipopolysaccharide biosynthesis protein RfbH [Bacteroides fragilis] | Bacterial outer membrane biogenesis; LPS O-antigen biosynthesis. | +2.2 |
| BF638R_RS17010  BF638R_3547 | cardiolipin synthase [Bacteroides fragilis] | Catalyzes the synthesis of cardiolipin (CL) (diphosphatidylglycerol) by specifically transferring a phosphatidyl group from CDP-diacylglycerol to phosphatidylglycerol (PG). | +2.1 |
| BF638R_RS17040  BF638R_3553 | biopolymer transporter ExbD [Bacteroides fragilis] | Involved in the TonB-dependent energy-dependent transport of various receptor-bound substrates.  This group of proteins are membrane bound transport proteins essential for ferric ion uptake in bacteria. The Pfam family consists of ExbD, and TolR which are involved in TonB-dependent transport of various receptor bound substrates including colicins. | +2.5 |
| BF638R_RS17050  BF638R_3555 | MotA/TolQ/ExbB proton channel family protein [Bacteroides fragilis] | Transmembrane helices of integral membrane proteins that appear to be involved in translocation of proteins or ions across a membrane. These proteins are probably proton channels. MotA is an essential component of the flagellar motor that uses a proton gradient to generate rotational motion in the flagellar [1, 2]. ExbB is part of the TonB-dependent transduction complex. The TonB complex uses the proton gradient across the inner bacterial membrane to transport large molecules across the outer bacterial membrane. | +6.4 |
| BF638R_RS18465  BF638R_3833 | TolC family protein [Bacteroides fragilis] | TolC and its homologues are involved in the export of chemically diverse molecules ranging from large protein toxins, such as alpha-hemolysin, to small toxic compounds, such as antibiotics. TolC family members thus play important roles in conferring pathogenic bacteria with both virulence and multidrug resistance. | -3.2 |
| BF638R_RS18545  BF638R_3849 | S8 family serine peptidase [Bacteroides fragilis] | The subtilisin-serine protease (SRSP) family hormone and pro-protein convertases (furin, PC1/3, PC2, PC4, PACE4, PC5/6, and PC7/7/LPC) act within the secretory pathway to cleave polypeptide precursors at specific basic sites, generating their biologically active forms. | -2.1 |
| BF638R_RS18670  BF638R_3873 | hypothetical protein [Bacteroides fragilis]  FimB/Mfa2 family fimbrial subunit |  | +5.5 |
| BF638R_RS18675  BF638R_3874 | membrane protein [Bacteroides fragilis] |  | +3.7 |
| BF638R_RS18685  BF638R_3876 | OmpA family protein [Bacteroides fragilis] |  | +3.5 |
| BF638R_RS18695  BF638R_3878 | FimB/Mfa2 family fimbrial subunit [Bacteroides fragilis] |  | +4.5 |
| BF638R_RS18710  BF638R_3882 | polymer-forming cytoskeletal protein [Bacteroides fragilis] | Polymer-forming cytoskeletal ;This is a family of bactofilins, a functionally diverse class of cytoskeletal, polymer-forming, proteins that is widely conserved among bacteria. In the example species C. crescentus, two bactofilins assemble into a membrane-associated laminar structure that shows cell-cycle-dependent polar localization and acts as a platform for the recruitment of a cell wall biosynthetic enzyme involved in polar morphogenesis.  Flagellar Assembly Protein A ;Members of this family include FapA (flagellar assembly protein A), found in Vibrio vulnificus. The synthesis of flagella allows bacteria to respond to chemotaxis by facilitating motility. | +5.9 |
| BF638R_RS19115  BF638R_3957 | M13 family metallopeptidase [Bacteroides fragilis] | The M13 family includes neprilysin (neutral endopeptidase, NEP, enkephalinase, CD10, CALLA, 3.4.24.11), endothelin-converting enzyme I (ECE-1, 3.4.24.71), erythrocyte surface antigen KELL (ECE-3), phosphate-regulating gene on the X chromosome (PHEX), soluble secreted endopeptidase (SEP), and damage-induced neuronal endopeptidase (DINE)/X-converting enzyme (XCE). These proteins consist of a short N-terminal cytoplasmic domain, a single transmembrane helix, and a larger C-terminal extracellular domain containing the active site. | +3.5 |
| BF638R_RS19360  BF638R_3999 | efflux RND transporter permease subunit [Bacteroides fragilis] | AcrB | +5 |
| BF638R_RS19365  BF638R_4000 | efflux RND transporter periplasmic adaptor subunit [Bacteroides fragilis] | AcrA | +9.1 |
| BF638R_RS20450  BF638R_4221 | M28 family peptidase [Bacteroides fragilis] | This domain is found in metallopeptidases belonging to the MEROPS peptidase family M28 (aminopeptidase Y, clan MH) and in non-peptidase homologues such as transferrin receptor proteins. Members containing this domain, also contain a transferrin receptor-like dimerisation domain (IPR007365) and a protease-associated PA domain (IPR003137). | +2.7 |
| BF638R_RS20490  BF638R_4229 | calcium-translocating P-type ATPase. PMCA-type [Bacteroides fragilis] | P-type ATPase responsible for translocating calcium ions across the plasma membrane of eukaryotes [1], out of the cell. In humans and mice, at least, there are multiple isoforms of the PMCA pump with overlapping but not redundant functions. The calcium P-type ATPases have been characterized as Type IIB based on a phylogenetic analysis which distinguishes this group from the Type IIA SERCA calcium pump | +3.9 |
| BF638R_RS20505  BF638R_4232 | Na/Pi cotransporter family protein [Bacteroides fragilis] | This family consists of sodium-dependent phosphate transport proteins of the solute carrier family SLC34A. It includes mammalian type II renal Na+/Pi-cotransporters and other proteins from lower eukaryotes and bacteria, some of which are also Na+/Pi-cotransporters. In kidneys these proteins may be involved in actively transporting phosphate into cells via Na+ cotransport in the renal brush border membrane | +5.2 |
| BF638R_RS20875  BF638R_4310 | protein translocase subunit SecDF [Bacteroides fragilis] | protein-export membrane protein SecD. This SecD and SecF proteins are part of the multimeric protein export complex comprising SecA, D, E, F, G, Y, and YajC. SecD and SecF are required to maintain a proton motive force. | +6 |
| BF638R_RS21135  BF638R_4366 | 6-bladed beta-propeller [Bacteroides fragilis] |  | +2.9 |
| BF638R_RS21330  BF638R_4407 | OmpA family protein [Bacteroides fragilis] |  | +5.9 |
| BF638R_RS21815  BF638R_4499 | succinate dehydrogenase/fumarate reductase cytochrome b subunit [Bacteroides fragilis] | succinate:quinone oxidoreductase (SQR)-like Type B subfamily 2, transmembrane subunit; composed of proteins with similarity to the SQRs of Geobacter metallireducens and Corynebacterium glutamicum. SQR catalyzes the oxidation of succinate to fumarate coupled to the reduction of quinone to quinol. SQR is composed of a flavoprotein catalytic subunit, an iron-sulfur protein and one or two hydrophobic transmembrane subunits. Members of this subfamily are classified as Type B as they contain one transmembrane subunit **and two heme groups**. The heme and quinone binding sites reside in the transmembrane subunit. The transmembrane subunit of members of this subfamily is also called Sdh cytochrome b558 subunit based on the Bacillus subtilis protein. The structural arrangement allows efficient electron transfer between the catalytic subunit, through iron-sulfur centers, and the transmembrane subunit containing the electron acceptor (quinone). The reversible reduction of quinone is an essential feature of respiration, allowing transfer of electrons between respiratory complexes. Proteins in this subfamily from G. metallireducens and G. sulfurreducens are bifunctional enzymes with SQR and QFR activities. | +11.5 |
| BF638R_RS21840  BF638R_4504 | TonB-dependent receptor [Bacteroides fragilis] |  | -32.2 |

**7.2. Protein synthesis (35/237):**

| **Data base entry** | **description** |  | **-fold up (+) or downregulation (-) in resistant 638R *nimA*** |
| --- | --- | --- | --- |
| BF638R_RS02660  BF638R_0559 | 50S ribosomal protein L19 [Bacteroides fragilis] |  | +5.7 |
| BF638R_RS04675  BF638R_0990 | 50S ribosomal protein L27 [Bacteroides fragilis] |  | +2.1 |
| BF638R_RS05690  BF638R_1211 | 50S ribosomal protein L25/general stress protein Ctc. partial [Bacteroides fragilis] |  | +4.5 |
| BF638R_RS07910  BF638R_1699 | translation initiation factor IF-3 [Bacteroides fragilis] | In molecular biology, translation initiation factor IF-3 (gene infC) is one of the three factors required for the initiation of protein biosynthesis in bacteria. | +3.6 |
| BF638R_RS07915  BF638R_1700 | 50S ribosomal protein L35. partial [Bacteroides fragilis] |  | +7.3 |
| BF638R_RS07920  BF638R_1701 | 50S ribosomal protein L20 [Bacteroides fragilis] |  | +22.8 |
| BF638R_RS11730  BF638R_2470 | 50S ribosomal protein L33 [Bacteroides fragilis] |  | +5.9 |
| BF638R_RS11735  BF638R_2471 | 50S ribosomal protein L28 [Bacteroides fragilis] |  | +4.6 |
| BF638R_2567 | RluA family pseudouridine synthase [Bacteroides fragilis] | Pseudouridine synthases catalyse the isomerisation of uridine to pseudouridine (Psi) in a variety of RNA molecules, and may function as RNA chaperones. Pseudouridine is the most abundant modified nucleotide found in all cellular RNAs. RluA, RluB, RluC, RluD, RluE and RluF act on large ribosomal subunit. | +26.2 |
| BF638R_RS15135  BF638R_3164 | type B 50S ribosomal protein L31. partial [Bacteroides fragilis] |  | +2.5 |
| BF638R_RS17795  BF638R_3704 | 30S ribosomal protein S18. partial [Bacteroides fragilis] |  | +9.7 |
| BF638R_RS17820  BF638R_3711 | elongation factor G [Bacteroides fragilis] | Catalyzes the GTP-dependent ribosomal translocation step during translation elongation. During this step, the ribosome changes from the pre-translocational (PRE) to the post-translocational (POST) state as the newly formed A-site-bound peptidyl-tRNA and P-site-bound deacylated tRNA move to the P and E sites, respectively. Catalyzes the coordinated movement of the two tRNA molecules, the mRNA and conformational changes in the ribosome | +6.8 |
| BF638R_RS18605  BF638R_3862 | 50S ribosomal protein L13 [Bacteroides fragilis] |  | +7.6 |
| BF638R_RS18620  BF638R_3866 | pseudouridine synthase [Bacteroides fragilis] | RsuA | +9.,7 |
| BF638R_RS19430  BF638R_4013 | 50S ribosomal protein L17 [Bacteroides fragilis] |  | +8.9 |
| BF638R_RS19440  BF638R_4015 | 30S ribosomal protein S4. partial [Bacteroides fragilis] |  | +9.4 |
| BF638R_RS19450  BF638R_4017 | 30S ribosomal protein S13. partial [Bacteroides fragilis] |  | +7.9 |
| BF638R_RS19470  BF638R_4021 | 50S ribosomal protein L15 [Bacteroides fragilis] |  | +6.7 |
| BF638R_RS19490  BF638R_4025 | 50S ribosomal protein L6, partial [Bacteroides fragilis] |  | +6.8 |
| BF638R_RS19495  BF638R_4026 | 30S ribosomal protein S8. partial [Bacteroides fragilis] |  | +3.5 |
| BF638R_RS19500  BF638R_4027 | 30S ribosomal protein S14 [Bacteroides fragilis] |  | +8.2 |
| BF638R_RS19505  BF638R_4028 | 50S ribosomal protein L24 [Bacteroides fragilis] |  | +4.5 |
| BF638R_RS19510  BF638R_4029 | 50S ribosomal protein L5 [Bacteroides fragilis] |  | +3.3 |
| BF638R_RS19520  BF638R_4033 | 30S ribosomal protein S17 [Bacteroides fragilis] |  | +5.8 |
| BF638R_RS19530  BF638R_4034 | 50S ribosomal protein L16 [Bacteroides fragilis] |  | +13.2 |
| BF638R_RS19535  BF638R_4035 | 30S ribosomal protein S3 [Bacteroides fragilis] |  | +15.1 |
| BF638R_RS19540  BF638R_4036 | 50S ribosomal protein L22 [Bacteroides fragilis] |  | +8.9 |
| BF638R_RS19545  BF638R_4037 | 30S ribosomal protein S19. partial [Bacteroides fragilis] |  | +12.7 |
| BF638R_RS19550  BF638R_4038 | 50S ribosomal protein L2 [Bacteroides fragilis] |  | +12.8 |
| BF638R_RS19560  BF638R_4040 | 50S ribosomal protein L4 [Bacteroides fragilis] |  | +2.4 |
| BF638R_RS19570  BF638R_4042 | 30S ribosomal protein S10. partial [Bacteroides fragilis] |  | +3.5 |
| BF638R_RS19580  BF638R_4044 | 30S ribosomal protein S7 [Bacteroides fragilis] |  | +5 |
| BF638R_RS19635  BF638R_4055 | 50S ribosomal protein L1 [Bacteroides fragilis] |  | +5.4 |
| BF638R_RS19695  BF638R_4063 | 30S ribosomal protein S21. partial [Bacteroides fragilis] |  | +2.2 |
| BF638R_RS21025  BF638R_4341 | 30S ribosomal protein S15. partial [Bacteroides fragilis] |  | +7.1 |

**7.3. DNA and RNA integrity (5/237):**

| **Data base entry** | **description** |  | **-fold up (+) or downregulation (-) in resistant 638R *nimA*** |
| --- | --- | --- | --- |
| BF638R_RS04760  BF638R_1010 | class I SAM-dependent rRNA methyltransferase [Bacteroides fragilis] | class I SAM-dependent rRNA methyltransferase catalyzes the methylation of one or more specific ribosomal RNA residues using S-adenosyl-L-methionine (SAM or AdoMet) as the methyl donor | +2.8 |
| BF638R_RS07305  BF638R_1564 | Rne/Rng family ribonuclease [Bacteroides fragilis] | This model describes ribonuclease G (formerly CafA, cytoplasmic axial filament protein A), the N-terminal domain of ribonuclease E in which ribonuclease activity resides, and related proteins. In E. coli, both RNase E and RNase G have been shown to play a role in the maturation of the 5' end of 16S RNA. The C-terminal half of RNase E (excluded from the seed alignment for this model) lacks ribonuclease activity but participates in mRNA degradation by organizing the degradosome. | +4 |
| BF638R_RS07310  BF638R_1566 | integration host factor subunit beta. partial [Bacteroides fragilis] | One of the 2 subunits of integration host factor (IHF), a specific DNA-binding protein that functions in genetic recombination as well as in transcriptional and translational control. | +3.8 |
| BF638R_RS16460  BF638R_3432 | ribonuclease Y [Bacteroides fragilis] | Endoribonuclease that initiates mRNA decay. Known from Bacillus subtilis. | +4.6 |
| BF638R_RS20850  BF638R_4305 | HU family DNA-binding protein. partial [Bacteroides fragilis] | HU is a small (10 kDa) bacterial histone-like protein that resembles the eukaryotic Histone H2B. HU acts similarly to a histone by inducing negative supercoiling into circular DNA with the assistance of topoisomerase. The protein has been implicated in DNA replication, recombination, and repair. HU binds non-specifically to dsDNA with low affinity but binds to altered DNA—such as junctions, nicks, gaps, forks, and overhangs—with high affinity. | +2.8 |

**7.4. Transcriptional regulators (9/237):**

| **Data base entry** | **description** |  | **-fold up (+) or downregulation (-) in resistant 638R *nimA*** |
| --- | --- | --- | --- |
| BF638R_RS05445  BF638R_1162 | SIR2 family protein. partial [Bacteroides fragilis] | The yeast SIR protein complex has been implicated in transcription silencing and suppression of recombination. The Sir complex represses transcription at telomeres, mating-type loci, and ribosomal DNA. | +2.1 |
| BF638R_RS05050  BF638R_1075 | UpxZ family transcription anti-terminator antagonist [Bacteroides fragilis] | Structural domains comprising this superfamily share the Structure of the family transcription anti-terminator antagonists UpxZ, which inhibit transcription of heterologous capsular polysaccharide loci in Bacteroides species by interfering with the action of the UpxY family of transcription anti-terminators. As antagonists of polysaccharide locus-specific UpxY transcription anti-terminators, the UpxZ proteins exert a hierarchical level of regulation, insuring that only one of the multiple phase-variable capsular polysaccharide loci per cell characteristic of this genus is transcribed at a time | -8.7 |
| BF638R_RS06680  BF638R_1434 | UpxZ family transcription anti-terminator antagonist [Bacteroides fragilis] | See above. | -2.1 |
| BF638R_RS07030  BF638R_1505 | helix-turn-helix domain-containing protein [Bacteroides fragilis] |  | +3.6 |
| BF638R_RS08730  BF638R_1863 | UpxZ family transcription anti-terminator antagonist [Bacteroides fragilis] |  | -6.2 |
| BF638R_RS10680  BF638R_2263 | Crp/Fnr family transcriptional regulator [Bacteroides fragilis] | Crp/Fnr regulators are global transcriptional regulators widely distributed in bacteria. The characteristic structure of Crp/Fnr is a C-terminal helix-turn-helix (HTH) motif that fits the DNA major groove and an N-terminal nucleotide binding domain | +2.4 |
| BF638R_RS15890  BF638R_3312 | TetR/AcrR family transcriptional regulator [Bacteroides fragilis] | The TetR family of regulators (TFRs) is a large and important family of one-component signal transduction systems. While members of this family are best known for their roles as regulators of antibiotic efflux pumps, this in fact describes a minority of their functional roles. Indeed, characterized members are known to regulate numerous aspects of bacterial physiology and to interact with a vast array of ligands | +2.1 |
| BF638R_RS16730  BF638R_3488 | UpxZ family transcription anti-terminator antagonist [Bacteroides fragilis] | See above. | +3.4 |
| BF638R_RS17805  BF638R_3706 | MarR family transcriptional regulator [Bacteroides fragilis] | Members of the multiple antibiotic resistance regulator (MarR) family of transcription factors are critical for bacterial cells to respond to chemical signals and to convert such signals into changes in gene activity. Obligate dimers belonging to the winged helix-turn-helix protein family, they are critical for regulation of a variety of functions. Inactivation of MarR gene homologs increases susceptibility to antimicrobials in Bacteroides fragilis. Braz J Microbiol. 2018 Jan-Mar;49(1):200-206. | +4.9 |

**7.5. Antimicrobial resistance-related (11/237):**

| **Data base entry** | **description** |  | **-fold up (+) or downregulation (-) in resistant 638R *nimA*** |
| --- | --- | --- | --- |
| BF638R_RS00810  BF638R_0178 | ABC transporter ATP-binding protein [Bacteroides fragilis] |  | +11.5 |
| BF638R_RS02635  BF638R_0554 | efflux RND transporter periplasmic adaptor subunit [Bacteroides fragilis] |  | +8.7 |
| BF638R_RS04280  BF638R_0908 | aminoglycoside phosphotransferase family protein [Bacteroides fragilis] | The aminoglycoside phosphotransferases inactivate aminoglycoside antibiotics via phosphorylation. | +2.1 |
| BF638R_RS10850  BF638R_2297 | efflux RND transporter periplasmic adaptor subunit [Bacteroides fragilis] | RND (Resistance-Nodulation-Division) family transporters are widespread especially among Gram-negative bacteria, and catalyze the active efflux of many antibiotics and chemotherapeutic agents. They have very large periplasmic domains, and form tripartite complexes with outer membrane channels and periplasmic adaptor proteins. | +3.8 |
| BF638R_RS10855  BF638R_2298 | efflux RND transporter permease subunit [Bacteroides fragilis] | See above | +4.8 |
| BF638R_RS11550  BF638R_2435 | efflux RND transporter periplasmic adaptor subunit [Bacteroides fragilis] | See above | +9.7 |
| BF638R_RS11555  BF638R_2436 | efflux RND transporter permease subunit [Bacteroides fragilis] | See above | +8.6 |
| BF638R_RS12530  BF638R_2622 | MBL fold metallo-hydrolase [Bacteroides fragilis] | Members of the MBL-fold metallohydrolase superfamily are mainly hydrolytic enzymes which carry out a variety of biological functions. The class B metal beta-lactamases (MBLs) for which this fold was named perform only a small fraction of the activities included in this superfamily. | +15 |
| BF638R_RS14315  BF638R_2994 | TolC family protein [Bacteroides fragilis] | TolC and its homologues are involved in the export of chemically diverse molecules ranging from large protein toxins, such as alpha-hemolysin, to small toxic compounds, such as antibiotics. TolC family members thus play important roles in conferring pathogenic bacteria with both virulence and multidrug resistance. | +4.7 |
| BF638R_RS15185  BF638R_3174 | efflux RND transporter periplasmic adaptor subunit [Bacteroides fragilis] | RND (Resistance-Nodulation-Division) family transporters are widespread especially among Gram-negative bacteria, and catalyze the active efflux of many antibiotics and chemotherapeutic agents. They have very large periplasmic domains, and form tripartite complexes with outer membrane channels and periplasmic adaptor proteins. | +8.4 |
| BF638R_RS15190  BF638R_3176 | efflux RND transporter permease subunit [Bacteroides fragilis] | See above. | +2.1 |

**7.6. Energy metabolism (11/237):**

| **Data base entry** | **description** |  | **-fold up (+) or downregulation (-) in resistant 638R *nimA*** |
| --- | --- | --- | --- |
| BF638R_RS00125  BF638R_0025 | acetyl-CoA hydrolase/transferase family protein [Bacteroides fragilis] | This enzyme belongs to the family of hydrolases, specifically those acting on thioester bonds. The systematic name is CoA thiol esterase. This enzyme participates in pyruvate metabolism. | +2 |
| BF638R_RS06275  BF638R_1338 | pyruvate formate lyase-activating protein [Bacteroides fragilis] | The activation of pyruvate formate-lyase (PFL) by pyruvate formate-lyase activating enzyme (PFL-AE) involves formation of a specific glycyl radical on PFL by the PFL-AE in a reaction requiring S-adenosylmethionine (AdoMet). | -10.8 |
| BF638R_RS06280  BF638R_1339 | formate C-acetyltransferase [Bacteroides fragilis] | Formate C-acetyltransferase (pyruvate formate lyase is an enzyme. Pyruvate formate lyase is found in Escherichia coli and other organisms. It helps regulate anaerobic glucose metabolism. Using radical non-redox chemistry, it catalyzes the reversible conversion of pyruvate and coenzyme-A into formate and acetyl-CoA. | -5.1 |
| BF638R_RS10070  BF638R_2137 | NADH:ubiquinone reductase (Na(+)-transporting) subunit B [Bacteroides fragilis] | The sodium -pumping NADH: ubiquinone oxidoreductase (Na+-NQR) is the main ion pump and the primary entry site for electrons into the respiratory chain of many different types of pathogenic bacteria. This enzymatic complex creates a transmembrane gradient of sodium that is used by the cell to sustain ionic homeostasis, nutrient transport, ATP synthesis, flagellum rotation and other essential processes. | +8.5 |
| BF638R_RS10075  BF638R_2138 | Na(+)-translocating NADH-quinone reductase subunit C [Bacteroides fragilis] | See above. | +5.5 |
| BF638R_RS10090  BF638R_2141 | NADH:ubiquinone reductase (Na(+)-transporting) subunit F [Bacteroides fragilis] | See above. | +3.6 |
| BF638R_RS12250  BF638R_2565 | Pyruvate, phosphate dikinase [Bacteroides fragilis] | Pyruvate phosphate dikinase (PPDK) is an enzyme that catalyzes the inter-conversion of adenosine triphosphate (ATP), phosphate (Pi), and pyruvate with adenine monophosphate (AMP), pyrophosphate (PPi), and phosphoenolpyruvate (PEP) in the presence of magnesium and potassium/sodium ions (Mg2+ and K+/Na+). | +2.1 |
| BF638R_RS12485  BF638R_2612 | electron transport complex subunit RsxC [Bacteroides fragilis] | Part of a membrane complex involved in electron transport. Required to maintain the reduced state of SoxR. | +8.4 |
| BF638R_RS17935  BF638R_3733 | 4Fe-4S dicluster domain-containing protein [Bacteroides fragilis] | Hydrogenase! | +5.9 |
| BF638R_RS17945  BF638R_3735 | [FeFe] hydrogenase H-cluster radical SAM maturase HydG [Bacteroides fragilis] | “Fe-S maturase” https://pubs.rsc.org/en/content/articlehtml/2020/sc/d0sc04216a | +3.2 |
| BF638R_RS20430  BF638R_4217 | butyrate kinase [Bacteroides fragilis] | Butyrate kinase is an enzyme that facilitates the formation of butyryl-CoA by phosphorylating butyrate in the presence of ATP to form butyryl phosphate. The final steps in butyrate synthesis by anaerobic bacteria can occur via butyrate kinase and phosphotransbutyrylase or via butyryl-CoA:acetate CoA-transferase, the latter providing the dominant route for butyrate formation in human colonic bacteria. | +10.9 |

**7.7. (N-)Sugar metabolism (28/237):**

| **Data base entry** | **description** |  | **-fold up (+) or downregulation (-) in resistant 638R *nimA*** |
| --- | --- | --- | --- |
| BF638R_RS00570  BF638R_0129 | mannonate dehydratase [Bacteroides fragilis] | This enzyme participates in pentose and glucuronate interconversions. | +2.8 |
| BF638R_RS00790  BF638R_0174 | glycosyltransferase family 8 protein [Bacteroides fragilis] | These enzymes catalyse the transfer of sugar moieties from activated donor molecules to specific acceptor molecules, forming glycosidic bonds. A classification of glycosyltransferases using nucleotide diphospho-sugar, nucleotide monophospho-sugar and sugar phosphates | +3 |
| BF638R_RS03485  BF638R_0728 | GH92 family glycosyl hydrolase [Bacteroides fragilis] | Members of this family are alpha-1,2-mannosidases, enzymes which remove alpha-1,2-linked mannose residues from Man(9)(GlcNAc)(2) by hydrolysis. They are critical for the maturation of N-linked oligosaccharides and ER-associated degradation. | +6.3 |
| BF638R_RS03715  BF638R_0778 | UDP-glucose dehydrogenase [Bacteroides fragilis] | The protein encoded by this gene converts UDP-glucose to UDP-glucuronate and thereby participates in the biosynthesis of glycosaminoglycans. | -9 |
| BF638R_RS03725  BF638R_0780 | CDP-glucose 4,6-dehydratase [Bacteroides fragilis] | CDP-D-glucose 4,6-dehydratase catalyzes the first irreversible step in the synthesis of this 3,6-dideoxysugar by converting CDP-D-glucose to CDP-4-keto-6-deoxyglucose via an NAD+ -dependent intramolecular oxidation-reduction reaction. | -5 |
| BF638R_RS03760  BF638R_0789 | polysaccharide biosynthesis protein [Bacteroides fragilis] |  | -5.5 |
| BF638R_RS04280  BF638R_0908 | aminoglycoside phosphotransferase family protein [Bacteroides fragilis] | The aminoglycoside phosphotransferases inactivate aminoglycoside antibiotics via phosphorylation. | +2.1 |
| BF638R_RS04625  BF638R_0979 | glycogen debranching enzyme N-terminal domain-containing protein [Bacteroides fragilis] | This entry represents the central domain of the glycogen debranching enzyme AGL. AGL is a multifunctional enzyme acting as 1,4-alpha-D-glucan:1,4-alpha-D-glucan 4-alpha-D-glycosyltransferase and amylo-1,6-glucosidase in glycogen degradation | +2.9 |
| BF638R_RS05080  BF638R_1081 | UDP-N-acetyl-D-mannosamine dehydrogenase [Bacteroides fragilis] | Catalyzes the four-electron oxidation of UDP-N-acetyl-D-mannosamine (UDP-ManNAc), reducing NAD+ and releasing UDP-N-acetylmannosaminuronic acid (UDP-ManNAcA). This enzyme participates in acetamido sugar biosynthesis in bacteria and archaea. | -7.9 |
| BF638R_RS05105  BF638R_1086 | DegT/DnrJ/EryC1/StrS family aminotransferase [Bacteroides fragilis] | This entry represents the 3-amino-5-hydroxybenzoic acid synthase family (AHBA_syn) that are probably all pyridoxal-phosphate-dependent aminotransferase enzymes with a variety of molecular functions. Members of the family have the same structural fold as members of the pyridoxal phosphate (PLP)-dependent aspartate aminotransferase superfamily. | -2.3 |
| BF638R_RS08060  BF638R_1728 | exo-alpha-sialidase [Bacteroides fragilis] | Neuraminidase. Exo-α-sialidase is a glycoside hydrolase that cleaves the glycosidic linkages of neuraminic acids | +2 |
| BF638R_RS08080  BF638R_1732 | glycoside hydrolase family 2 protein [Bacteroides fragilis] |  | -7.5 |
| BF638R_RS08085  BF638R_1733 | family 20 glycosylhydrolase [Bacteroides fragilis] | Glycoside hydrolases are a widespread group of enzymes that hydrolyse the glycosidic bond between two or more carbohydrates, or between a carbohydrate and a non-carbohydrate moiety. | +7.9 |
| BF638R_RS08765  BF638R_1870 | DegT/DnrJ/EryC1/StrS family aminotransferase [Bacteroides fragilis] | See above. | -8.4 |
| BF638R_RS08805  BF638R_1878 | UDP-N-acetylglucosamine 2-epimerase (non-hydrolyzing) [Bacteroides fragilis] | This bacterial enzyme catalyses the reversible interconversion of UDP-GlcNAc and UDP-ManNAc. The latter is used in a variety of bacterial polysaccharide biosyntheses. | -3.5 |
| BF638R_RS12400  BF638R_2595 | CDP-glucose 4.6-dehydratase [Bacteroides fragilis] | CDP-D-glucose 4,6-dehydratase catalyzes the first irreversible step in the synthesis of this 3,6-dideoxysugar by converting CDP-D-glucose to CDP-4-keto-6-deoxyglucose via an NAD+ -dependent intramolecular oxidation-reduction | +2.5 |
| BF638R_RS12415  BF638R_2598 | lipopolysaccharide biosynthesis protein RfbH [Bacteroides fragilis] | Bacterial outer membrane biogenesis; LPS O-antigen biosynthesis. | +2.4 |
| BF638R_RS14875  BF638R_3114 | beta-N-acetylhexosaminidase [Bacteroides fragilis] | n general, hexosaminidases describe enzymes that cleave the glycosidic linkage of 2-acetamido-2-deoxy-β-d-glycosides. This group of enzymes includes, for example, lysozymes, chitinases, chitobioses, hyaluronidases, O-GlcNAcase, and others. | +7.4 |
| BF638R_RS14935  BF638R_3126 | type I pullulanase [Bacteroides fragilis] | Pullulanase is a specific kind of glucanase, an amylolytic exoenzyme, that degrades pullulan. | +5 |
| BF638R_RS15225  BF638R_3183 | family 10 glycosylhydrolase [Bacteroides fragilis] | Glycoside hydrolase family 10 CAZY GH_10 comprises enzymes with a number of known activities; xylanase); endo-1,3-beta-xylanase; cellobiohydrolase. These enzymes were formerly known as cellulase family F. | +7.6 |
| BF638R_RS15330  BF638R_3203 | GH32 C-terminal domain-containing protein [Bacteroides fragilis] | This family contains enzymes that hydrolyze fructose containing polysaccharides such as inulinases and exo-inulinases, levanases and β-2,6-fructan 6-levanbiohydrolases, fructan β-(2,1)-fructosidase/1-exohydrolase or fructan β-(2,6)-fructosidase/6-exohydrolases, as well as enzymes displaying transglycosylating activities | +11.8 |
| BF638R_RS15455  BF638R_3228 | alpha-L-fucosidase [Bacteroides fragilis] | Alpha-L-fucosidase is responsible for hydrolyzing the alpha-1,6-linked fucose joined to the reducing-end N-acetylglucosamine of the carbohydrate moieties of glycoproteins. | -7.1 |
| BF638R_RS16410  BF638R_3422 | LUD domain-containing protein [Bacteroides fragilis] | Lactate utilizatin domain. https://www.ncbi.nlm.nih.gov/pmc/articles/PMC3924224/ | +2.6 |
| BF638R_RS16655  BF638R_3473 | glucose-1-phosphate thymidylyltransferase RfbA [Bacteroides fragilis] | Catalyzes the formation of dTDP-glucose, from dTTP and glucose 1-phosphate, as well as its pyrophosphorolysis. | +4.7 |
| BF638R_RS16695  BF638R_3481 | GDP-mannose 4.6-dehydratase [Bacteroides fragilis] | This enzyme forms the first step in the biosynthesis of GDP-alpha-D-rhamnose and GDP-beta-L-fucose. Belongs to the short-chain dehydrogenase/reductase enzyme family | +3.2 |
| BF638R_RS16720  BF638R_3486 | lipopolysaccharide biosynthesis protein RfbH [Bacteroides fragilis] | Bacterial outer membrane biogenesis; LPS O-antigen biosynthesis. | +2.2 |
| BF638R_RS17695  BF638R_3685 | family 20 glycosylhydrolase [Bacteroides fragilis] | Glycoside hydrolases are a widespread group of enzymes that hydrolyse the glycosidic bond between two or more carbohydrates, or between a carbohydrate and a non-carbohydrate moiety. | +2.5 |
| BF638R_RS18125  BF638R_3770 | polysaccharide biosynthesis protein [Bacteroides fragilis] |  | -5.7 |

**7.8. Peptidases (9/237):**

| **Data base entry** | **description** |  | **-fold up (+) or downregulation (-) in resistant 638R *nimA*** |
| --- | --- | --- | --- |
| BF638R_RS03560  BF638R_0745 | ATP-dependent zinc metalloprotease FtsH [Bacteroides fragilis] | Acts as a processive, ATP-dependent zinc metallopeptidase for both cytoplasmic and membrane proteins. Plays a role in the quality control of integral membrane proteins. | +6.3 |
| BF638R_RS04440  BF638R_0940 | S41 family peptidase [Bacteroides fragilis] | The C-terminal processing peptidase (CPP, EC 3.4.21.102) also known as tail-specific protease (tsp), the photosystem II D1 C-terminal processing protease (D1P), and other related S41 protease family members are present in this CD. The bacterial CCP-1 is believed to be important for the degradation of incorrectly synthesized proteins as well as protection from thermal and osmotic stresses. | +2.3 |
| BF638R_RS07635  BF638R_1641 | C10 family peptidase [Bacteroides fragilis] | Peptidase family C10 contains bacterial cysteine endopeptidases. | +26.2 |
| BF638R_RS12525  BF638R_2621 | S9 family peptidase [Bacteroides fragilis] | This domain covers the active site serine of the serine peptidases belonging to MEROPS peptidase family S9 (prolyl oligopeptidase family, clan SC). | +3.3 |
| BF638R_RS14645  BF638R_3066  FQ312004 | trypsin-like peptidase domain-containing protein [Bacteroides fragilis] |  | +3.3 |
| BF638R_RS15855  BF638R_3306 | signal peptide peptidase SppA [Bacteroides fragilis] | Signal peptide peptidase (SPP) is a membrane-bound enzyme that uses a serine/lysine catalytic dyad mechanism to cleave the remnant signal peptides in the cellular membrane and aids in protein secretion. | +2.4 |
| BF638R_RS18545  BF638R_3849 | S8 family serine peptidase [Bacteroides fragilis] | The subtilisin-serine protease (SRSP) family hormone and pro-protein convertases (furin, PC1/3, PC2, PC4, PACE4, PC5/6, and PC7/7/LPC) act within the secretory pathway to cleave polypeptide precursors at specific basic sites, generating their biologically active forms. | -2.1 |
| BF638R_RS19115  BF638R_3957 | M13 family metallopeptidase [Bacteroides fragilis] | he M13 family includes neprilysin (neutral endopeptidase, NEP, enkephalinase, CD10, CALLA, 3.4.24.11), endothelin-converting enzyme I (ECE-1, 3.4.24.71), erythrocyte surface antigen KELL (ECE-3), phosphate-regulating gene on the X chromosome (PHEX), soluble secreted endopeptidase (SEP), and damage-induced neuronal endopeptidase (DINE)/X-converting enzyme (XCE). These proteins consist of a short N-terminal cytoplasmic domain, a single transmembrane helix, and a larger C-terminal extracellular domain containing the active site. | +3.5 |
| BF638R_RS20450  BF638R_4221 | M28 family peptidase [Bacteroides fragilis] | This domain is found in metallopeptidases belonging to the MEROPS peptidase family M28 (aminopeptidase Y, clan MH) and in non-peptidase homologues such as transferrin receptor proteins. Members containing this domain, also contain a transferrin receptor-like dimerisation domain (IPR007365) and a protease-associated PA domain (IPR003137). | +2.7 |

**7.9. Other Proteins and enzymes (28/237):**

| **Data base entry** | **description** |  | **-fold up (+) or downregulation (-) in resistant 638R *nimA*** |
| --- | --- | --- | --- |
| BF638R_RS00100  BF638R_0020 | rubrerythrin family protein [Bacteroides fragilis] | Rubrerythrin (RBR) is a non-heme iron-containing metalloprotein involved in oxidative stress tolerance within anaerobic bacteria. Reduces peroxides | +4.4 |
| F638R_RS00535  BF638R_0122 | tyrosine-protein phosphatase [Bacteroides fragilis] | The classical protein tyrosine phosphatases (PTPs) can be categorized as receptor-like (R) or non-transmembrane (NT) proteins. | +3.7 |
| BF638R_RS01515  BF638R_0326 | 1-acyl-sn-glycerol-3-phosphate acyltransferase [Bacteroides fragilis] | Converts 1-acyl-sn-glycerol-3-phosphate (lysophosphatidic acid or LPA) into 1,2-diacyl-sn-glycerol-3-phosphate (phosphatidic acid or PA) by incorporating an acyl moiety at the sn-2 position of the glycerol backbone. FA synthesis | +4.9 |
| BF638R_RS02180  BF638R_0463 | TIGR00159 family protein [Bacteroides fragilis]  diadenylate cyclase CdaA | The recently identified second messenger cyclic di-AMP (c-di-AMP) is involved in several important cellular processes, such as cell wall metabolism, maintenance of DNA integrity, ion transport, transcription regulation, and allosteric regulation of enzyme function | +5.8 |
| BF638R_RS04175  BF638R_0886 | bifunctional UDP-3-O-[3-hydroxymyristoyl] N-acetylglucosamine deacetylase/3-hydroxyacyl-ACP dehydratase [Bacteroides fragilis] | Catalyzes the hydrolysis of UDP-3-O-myristoyl-N-acetylglucosamine to form UDP-3-O-myristoylglucosamine and acetate, the committed step in lipid A biosynthesis. | +3.5 |
| BF638R_RS04660  BF638R_0987 | bifunctional dihydroorotate dehydrogenase B NAD binding subunit/NADPH-dependent glutamate synthase [Bacteroides fragilis] | DHODH is one of three enzymes that catalyze the six enzymatic reactions needed for de novo synthesis of pyrimidine. DHODH catalyzes the conversion of dihydroorotate to orotic acid, which is then converted to uridine monophosphate, the RNA nucleotide essential for ribosome biogenesis. | +2.3 |
| BF638R_RS05040  BF638R_1072 | class I SAM-dependent methyltransferase [Bacteroides fragilis] | S-adenosyl methionine (SAM) is a universal biological cofactor that is found in all branches of life where it plays a critical role in the transfer of methyl groups to various biomolecules, including DNA, proteins and small-molecule secondary metabolites | -8.4 |
| BF638R_RS05930  BF638R_1262 | catalase [Bacteroides fragilis] |  | +2.1 |
| BF638R_RS07205  BF638R_1544 | GNAT family N-acetyltransferase [Bacteroides fragilis] | Enzymes in the Gcn5-related N-acetyltransferase (GNAT) superfamily are widespread and critically involved in multiple cellular processes ranging from antibiotic resistance to histone modification. While acetyl transfer is the most widely catalyzed reaction, recent studies have revealed that these enzymes are also capable of performing succinylation, condensation, decarboxylation, and methylcarbamoylation reactions. | -3.2 |
| BF638R_RS07345  BF638R_1573 | (4Fe-4S)-binding protein [Bacteroides fragilis] |  | +2.2 |
| BF638R_RS07505  BF638R_1612 | NAD(P)/FAD-dependent oxidoreductase [Bacteroides fragilis] |  | +2,8 |
| BF638R_RS07565  BF638R_1626 | acetyl-CoA carboxylase biotin carboxylase subunit [Bacteroides fragilis] | Acetyl-CoA carboxylase (ACC) is a biotin-dependent enzyme (that catalyzes the irreversible carboxylation of acetyl-CoA to produce malonyl-CoA through its two catalytic activities, biotin carboxylase (BC) and carboxyltransferase (CT). | +2.5 |
| BF638R_RS09035  BF638R_1925 | LemA family protein [Bacteroides fragilis] |  | +3.6 |
| BF638R_RS10770  BF638R_2281 | RelA/SpoT family protein [Bacteroides fragilis] | RelA/SpoT-homologue proteins (RHS) mediate the stringent response in bacteria which enables its metabolic adaptation under stress conditions. These enzymes synthesize the second messenger (p)ppGpp, a small molecule also known as 'alarmone', which is a regulatory metabolite of the stringent response, characterised by growth arrest and the modulation of gene expression in response to various nutritional stresses | +4 |
| BF638R_RS11675  BF638R_2458 | protein BatD [Bacteroides fragilis] | This family of proteins carries up to three membrane spanning regions and is involved in tolerance to oxygen in in Bacteroides species. See Mol. Microbiol. 32, 139-49, (1999)  Bacteroides aerotolerance. Membrane protein. | +4.2 |
| BF638R_RS11690  BF638R_2461 | VWA domain-containing protein [Bacteroides fragilis] | VWA domains are the predominant independent folding units within matrilins and mediate protein–protein interactions. | +3.3 |
| BF638R_RS12065  BF638R_2534 | ATP-binding protein [Bacteroides fragilis] |  | +7.5 |
| BF638R_RS12395  BF638R_2594 | thiamine pyrophosphate-binding protein [Bacteroides fragilis] |  | +2 |
| BF638R_RS12485  BF638R_2612 | electron transport complex subunit RsxC [Bacteroides fragilis] | Part of a membrane complex involved in electron transport. Required to maintain the reduced state of SoxR. | +8.4 |
| BF638R_RS12870  BF638R_2695 | phosphoribosylaminoimidazolecarboxamide formyltransferase [Bacteroides fragilis] | An enzyme that catalyzes the conversion of aminoimidazole-4-carboxamide ribonucleotide to 5-formyl-aminoimidazole-4-carboxamide ribonucleotide in the purine de novo synthesis pathway. Requires formyl-tetrahydrofolate. | -7.5 |
| BF638R_RS12895  BF638R_2701 | thioredoxin [Bacteroides fragilis] | See Paunkov et al. 2022! TrxC | -7.1 |
| BF638R_RS12905  BF638R_2703 | methionine synthase [Bacteroides fragilis] | Methionine synthase also known as MS, MeSe, MTR is responsible for the regeneration of methionine from homocysteine. | -10.3 |
| BF638R_RS14505  BF638R_3035 | histidinol dehydrogenase [Bacteroides fragilis] | In bacteria, fungi, and plants, HDH catalyzes the terminal step in the biosynthesis of histidine, a four-electron oxidation of l-histidinol to histidine. | -2.4 |
| BF638R_RS14520  BF638R_3038 | PaaI family thioesterase [Bacteroides fragilis] | PaaI thioesterases are members of the TE13 thioesterase family that catalyze the hydrolysis of thioester bonds between coenzyme A and phenylacetyl-CoA. | +2.5 |
| BF638R_RS15270  BF638R_3191 | ATP-binding protein [Bacteroides fragilis] |  | +19.1 |
| BF638R_RS18075  BF638R_3760 | FAD-dependent oxidoreductase [Bacteroides fragilis]  Note: HemY --> Protoporphyrinogen oxidase | Protoporphyrinogen oxidase. This enzyme oxidizes protoporphyrinogen IX to protoporphyrin IX, a precursor of heme and chlorophyll. | -5.3 |
| BF638R_RS18940  BF638R_3929 | peptidylprolyl isomerase [Bacteroides fragilis] | peptidylprolyl isomerase catalyzes the cis-trans isomerization of proline imidic peptide bonds in oligopeptides | +8.5 |
| BF638R_RS19595  BF638R_4047 | YtxH domain-containing protein [Bacteroides fragilis] | This family of proteins is found in bacteria. Proteins in this family are typically between 100 and 143 amino acids in length. The N-terminal region is the most conserved. Proteins is this family are functionally uncharacterized. | +10.4 |

**7.10. Hypothetical proteins and proteins with domains without known functions (35/237):**

| **Data base entry** | **description** |  | **-fold up (+) or downregulation (-) in resistant 638R *nimA*** |
| --- | --- | --- | --- |
| BF638R_RS00315  BF638R_0070 | DUF4861 domain-containing protein [Bacteroides fragilis] |  | +23.2 |
| BF638R_RS00330  BF638R_0073 | DUF2723 domain-containing protein [Bacteroides fragilis] |  | +4.4 |
| BF638R_RS01790  BF638R_0384 | DUF4982 domain-containing protein [Bacteroides fragilis] |  | +8.3 |
| BF638R_RS01845  BF638R_0395 | DUF3131 domain-containing protein [Bacteroides fragilis] |  | +3.5 |
| BF638R_RS03020  BF638R_0633 | hypothetical protein [Bacteroides fragilis] |  | +4.8 |
| BF638R_RS03445  BF638R_0720 | hypothetical protein [Bacteroides fragilis] |  | +3.6 |
| BF638R_RS03640  BF638R_0760 | DUF1573 domain-containing protein [Bacteroides fragilis] |  | +2.9 |
| BF638R_RS04560  BF638R_0965 | hypothetical protein [Bacteroides fragilis] |  | +3.1 |
| BF638R_RS04715  BF638R_1001 | hypothetical protein [Bacteroides fragilis] |  | +4.1 |
| BF638R_RS04735  BF638R_1005 | DUF4836 family protein [Bacteroides fragilis] |  | +5.3 |
| BF638R_RS04985  BF638R_1061 | DUF4837 family protein [Bacteroides fragilis] |  | +2.3 |
| BF638R_RS06000  BF638R_1279 | DUF4890 domain-containing protein [Bacteroides fragilis] | Periplasmic? | +11.5 |
| BF638R_RS06100  BF638R_1302 | DUF488 family protein [Bacteroides fragilis] |  | +2.2 |
| BF638R_RS06230  BF638R_1329 | DUF1735 domain-containing protein [Bacteroides fragilis] |  | +14.1 |
| BF638R_RS06235  BF638R_1330 | DUF1735 domain-containing protein [Bacteroides fragilis] |  | +4.7 |
| BF638R_RS06650  BF638R_1427 | hypothetical protein [Bacteroides fragilis] |  | +10.4 |
| BF638R_RS07650  BF638R_1644 | hypothetical protein [Bacteroides fragilis] |  | +2.4 |
| BF638R_RS07760  BF638R_1667 | hypothetical protein [Bacteroides fragilis] |  | +2.4 |
| BF638R_RS08445  BF638R_1805 | hypothetical protein [Bacteroides fragilis] |  | -2.4 |
| BF638R_RS08450  BF638R_1806 | hypothetical protein [Bacteroides fragilis] |  | -2.9 |
| BF638R_RS08685  BF638R_1854 | hypothetical protein [Bacteroides fragilis] |  | +10.1 |
| BF638R_RS09120  BF638R_1941 | hypothetical protein [Bacteroides fragilis] |  | -9.9 |
| BF638R_RS10465  BF638R_2219 | hypothetical protein [Bacteroides fragilis] |  | -29.4 |
| BF638R_RS10470  BF638R_2220 | DUF4903 domain-containing protein [Bacteroides fragilis] |  | -130.2 |
| BF638R_RS12945  BF638R_2714 | hypothetical protein [Bacteroides fragilis] |  | -4.3 |
| BF638R_RS14130  BF638R_2956 | hypothetical protein [Bacteroides fragilis] |  | +2.1 |
| BF638R_RS14595  BF638R_3053 | GGGtGRT protein, partial [Bacteroides fragilis] | This family of proteins is functionally uncharacterised. | -9.7 |
| BF638R_RS14745  BF638R_3087 | DUF4857 domain-containing protein [Bacteroides fragilis] |  | -103 |
| BF638R_RS14765  BF638R_3091 | hypothetical protein [Bacteroides fragilis] |  | -14 |
| BF638R_RS14775  BF638R_3093 | DUF4876 domain-containing protein [Bacteroides fragilis] |  | -26 |
| BF638R_RS16050  BF638R_3348 | hypothetical protein [Bacteroides fragilis] |  | +4.1 |
| BF638R_RS16335  BF638R_3408 | hypothetical protein [Bacteroides fragilis] |  | +10 |
| BF638R_RS16530  BF638R_3446 | hypothetical protein [Bacteroides fragilis] |  | -5.5 |
| BF638R_RS16615  BF638R_3465 | hypothetical protein [Bacteroides fragilis] |  | +3.8 |
| BF638R_RS19420  BF638R_4011 | hypothetical protein [Bacteroides fragilis] |  | +4.7 |
